# Supplementary material for: Atomic engineering of high-density isolated Co atoms on graphene with proximal-atom controlled reaction selectivity
Source: Nat Commun. 2018 Aug 23;9:3197. doi: 10.1038/s41467-018-05754-9 (PMC6107637; doi:10.1038/s41467-018-05754-9)
Supplement: Supplementary file 1 — Supplementary Information [file 41467_2018_5754_MOESM1_ESM.pdf]

Atomic Engineering of High-Density Isolated Co Atoms on Graphene with Proximal-Atom Controlled Reaction Selectivity

Yan et al.

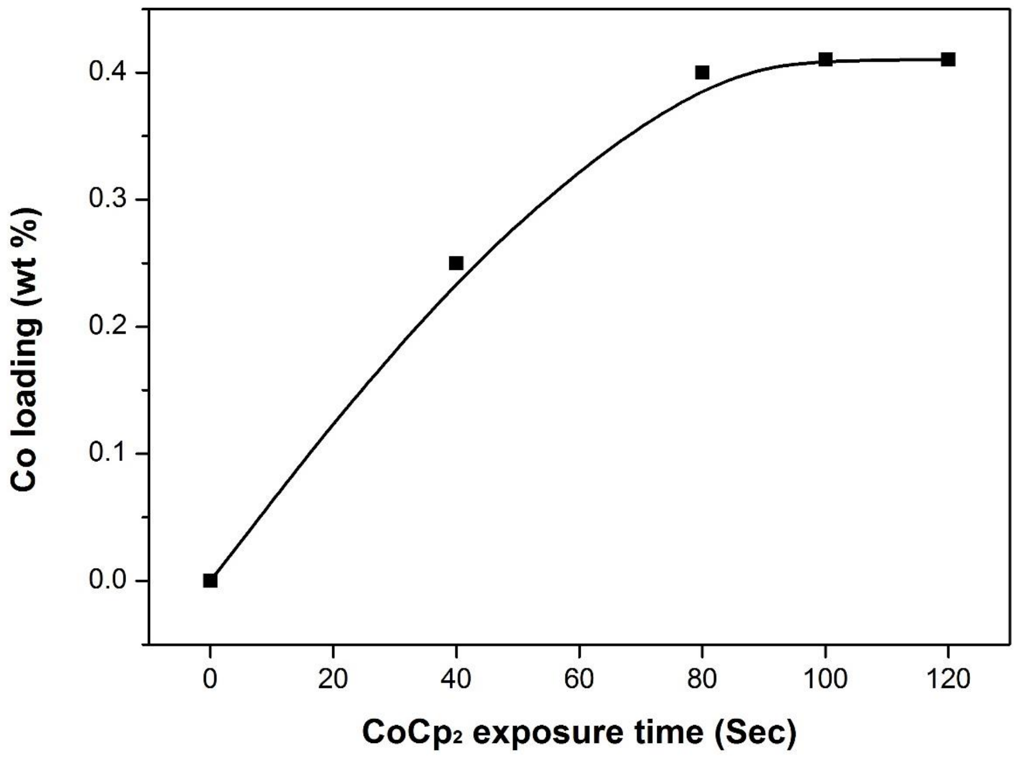

Supplementary Figure 1 │The Co loadings of Co_1/G with different $CoCp_2$ exposure time. The Co loadings of these catalysts were determined by ICP-AES.

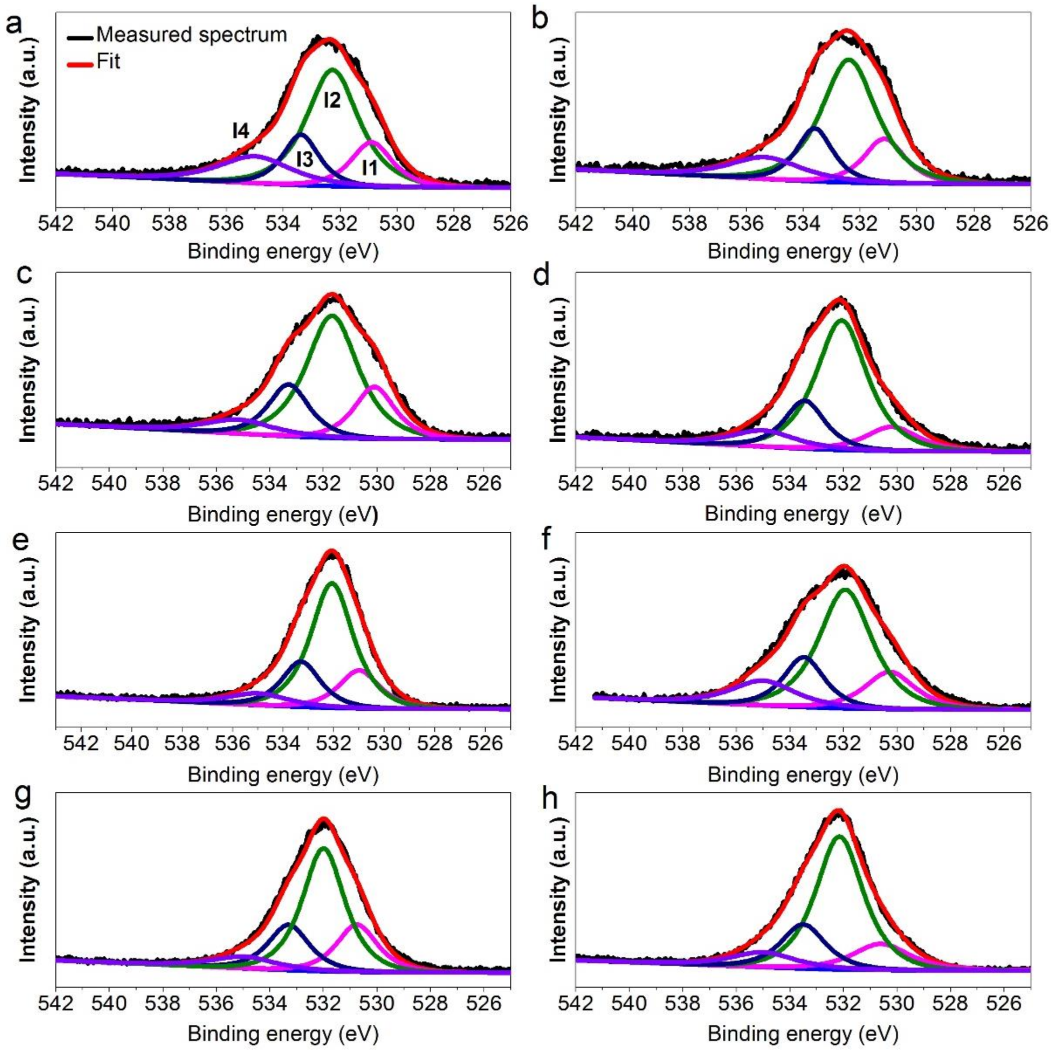

Supplementary Figure 2 | The evolution of oxygen content of graphene oxide. O1s XPS spectra of graphene oxide after one (a), two (b), three (c), four (d), five (e), six (f), seven (g) and eight (h), ALD cycles. The main components deconvolved from the O1s spectra are peaked around 531.08, 532.03, 533.43 and 534.7 eV, which can be assigned to C=O (oxygen double-bonded to aromatic carbon designated as **I1**), epoxide functional group (oxygen single-bonded to aliphatic carbon designated as **I2**), phenolic oxygen (designated as **I3**), chemisorbed water molecules (designated as **I4**) respectively, in line with the literature.[1, 2] Based on the XPS data, the epoxide functional group is identified as the dominant oxygen containing functional groups among all the samples prepared by ozone treatment. The content of epoxide functional group increases as the number of ALD (O_3 exposure) cycles increases, which accounts for about 65% of all oxygen groups.

Notes: The graphene here is just subjected to O_3 without exposure of sample to Co precursor.

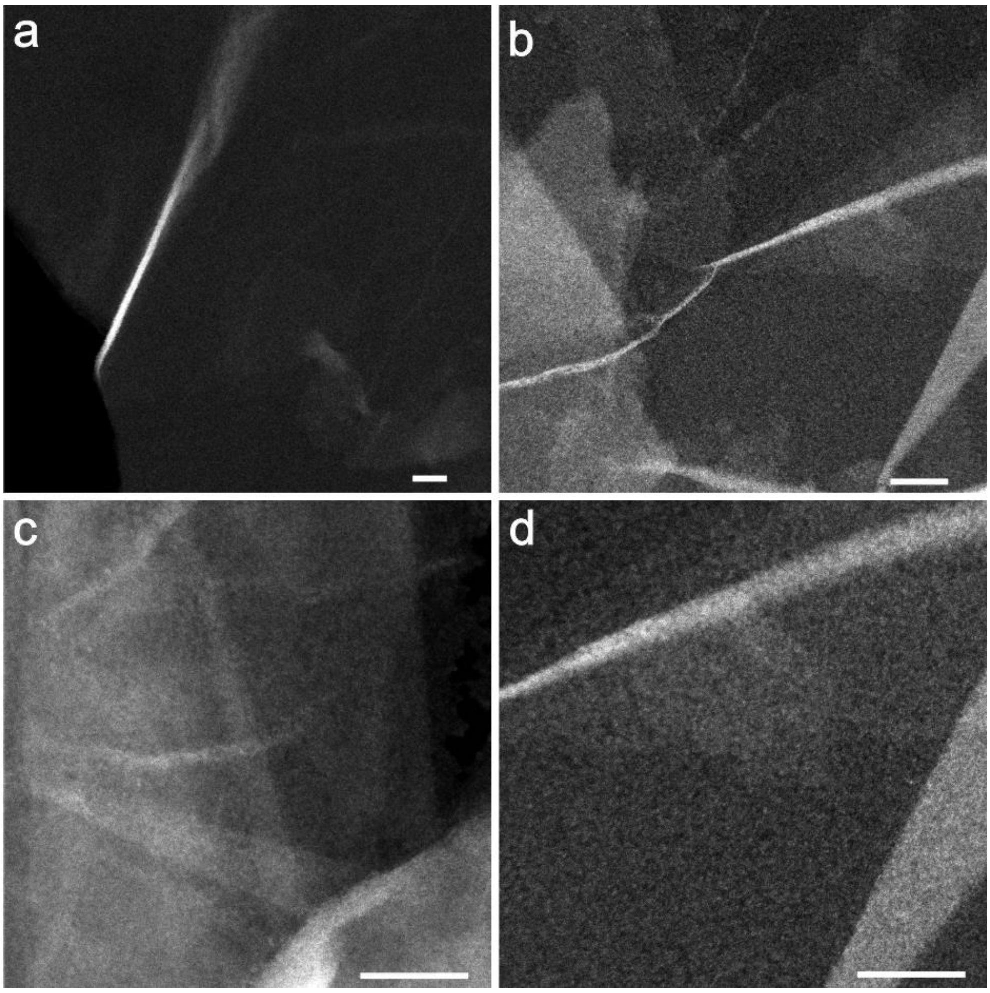

Supplementary Figure 3 │The morphologies of the Co_1/G SACs. The STEM-ADF images of Co_1/G-0.4 (a), Co_1/G-0.8 (b), Co_1/G-1.3 (c) and Co_1/G-2.0 (d) at low magnifications. Sacle bars, 50 nm (a-d).

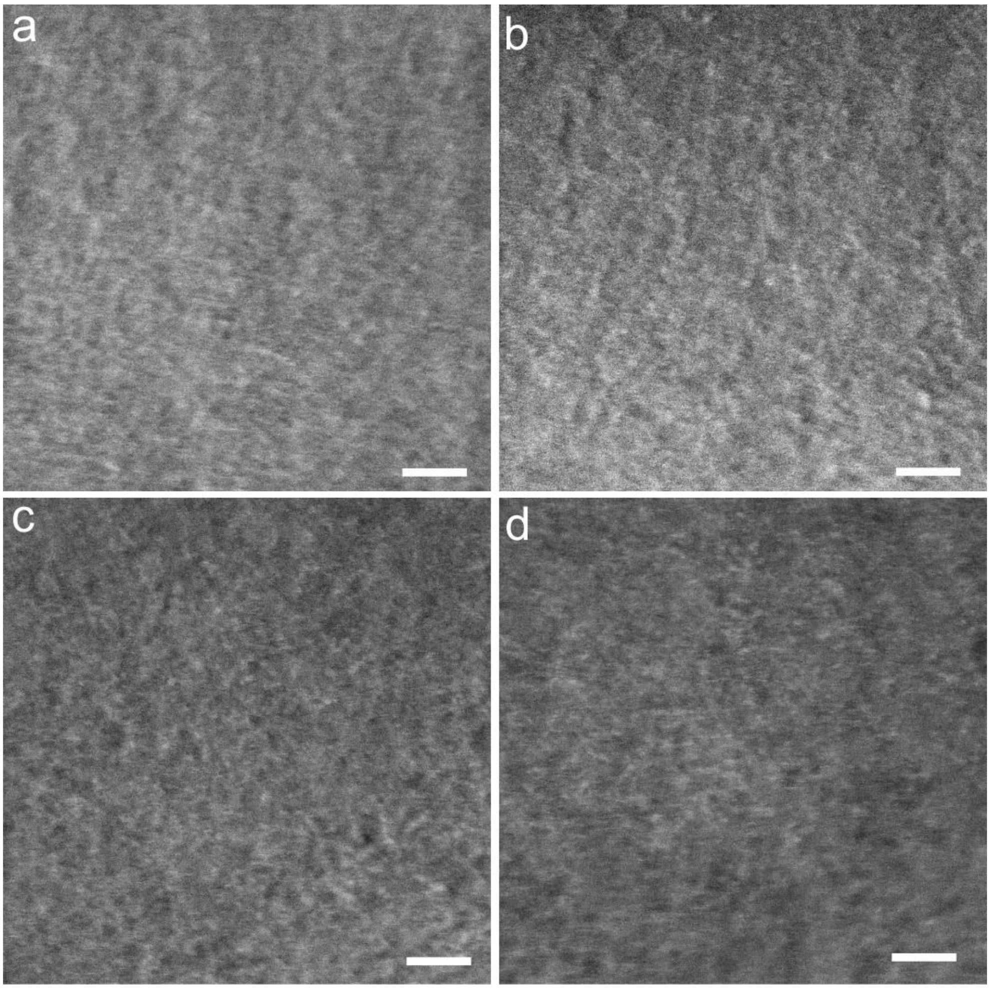

Supplementary Figure 4 │The morphologies of the graphene oxide support. STEM-ADF images of the reduced graphene oxide support at high magnifications (a-d). Scale bars, 2 nm (a-d).

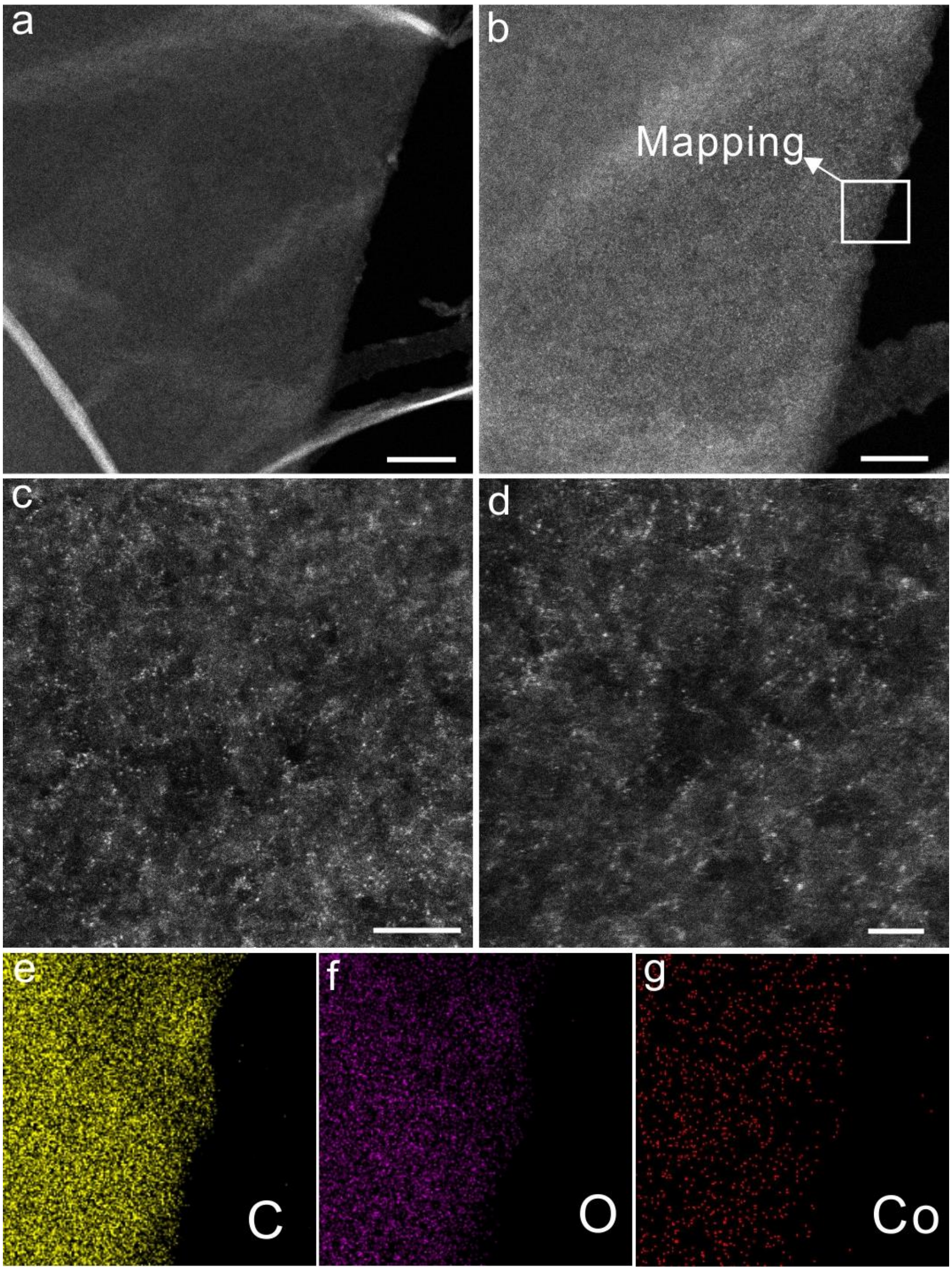

Supplementary Figure 5 │The morphology of the Co_1/G-0.8. Representative aberration-corrected STEM-ADF images of Co_1/G-0.8 in different spatial locations (a-d) and STEM EDS elemental mapping (e-g). Scale bars, 50 nm (a), 20 nm (b), 5 nm (c), 2 nm (d).

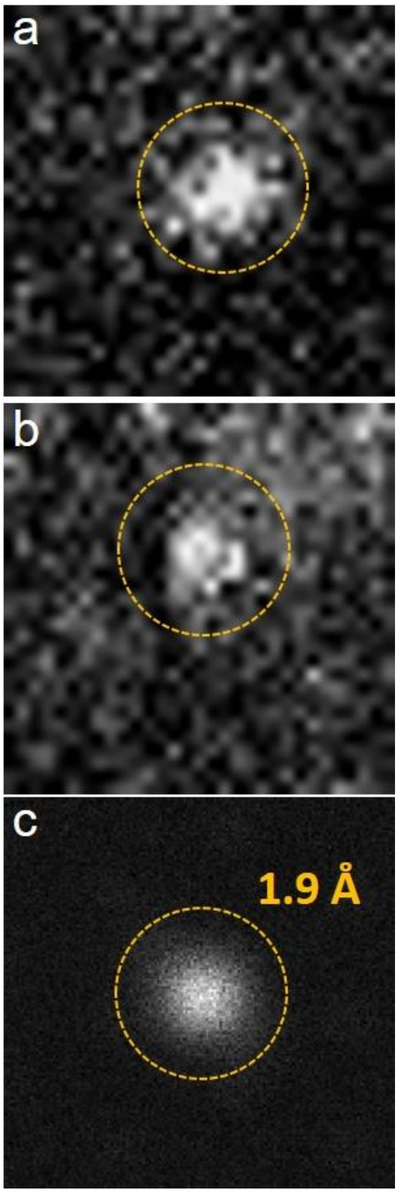

Supplementary Figure 6 | STEM-ADF images of Co atoms in comparison with the simulated image of Co atoms in graphene.

The atomic model used for simulation contains single Co atom anchored by four carbon atoms and two oxygen atoms on the surface of a four-layer graphene sample. The carbon and oxygen atoms are invisible in the images due to its much smaller atomic number compared to that of Co atoms.[3] Field of view (FOV): 8 Å (for all images). The simulation was performed using the QSTEM package (assuming an aberration-free probe and a source size of ~1.2 Å which produces a probe size of ~1 Å. The radius of the bright feature in experimental STEM image is determined to be 1.7-1.9 Å (a, b), close to the radius of the bright feature (1.9 Å) in the simulated image of a single Co atom. This further confirms that the bright features imaged in the Co_1 SACs can be assigned to atomically dispersed Co atoms rather than larger Co species such as dimers and trimmers, which would be explicitly resolved as such

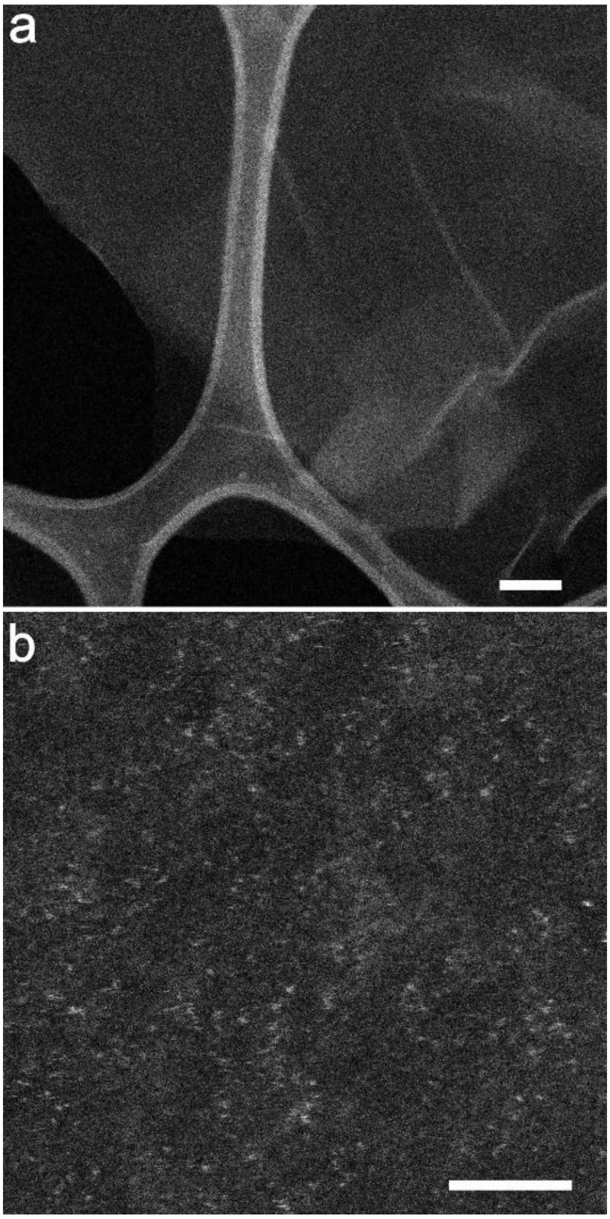

Supplementary Figure 7 | Representative aberration-corrected STEM-ADF images of Co_1/G-1.3 at different locations. Scale bars, 100 nm (a), 2 nm (b).

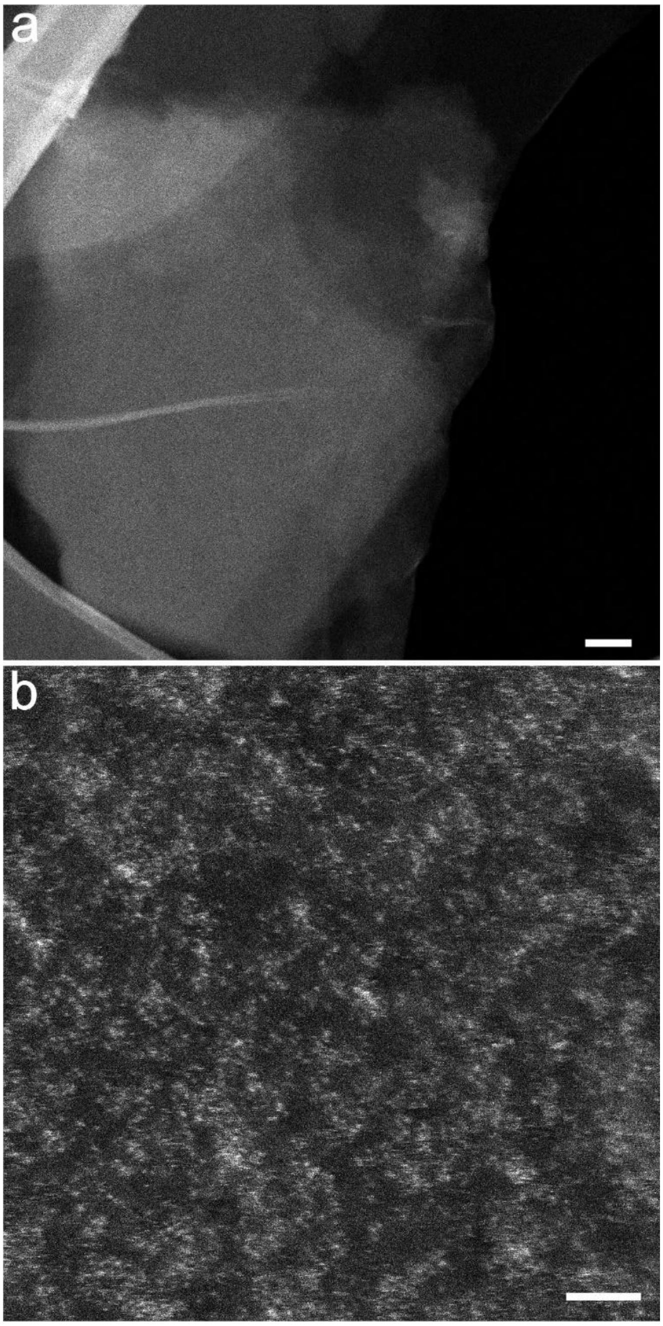

Supplementary Figure 8 | Representative aberration-corrected STEM-ADF images of Co_1/G-2.0 at different locations. Scale bars, 50 nm (a), 2 nm (b).

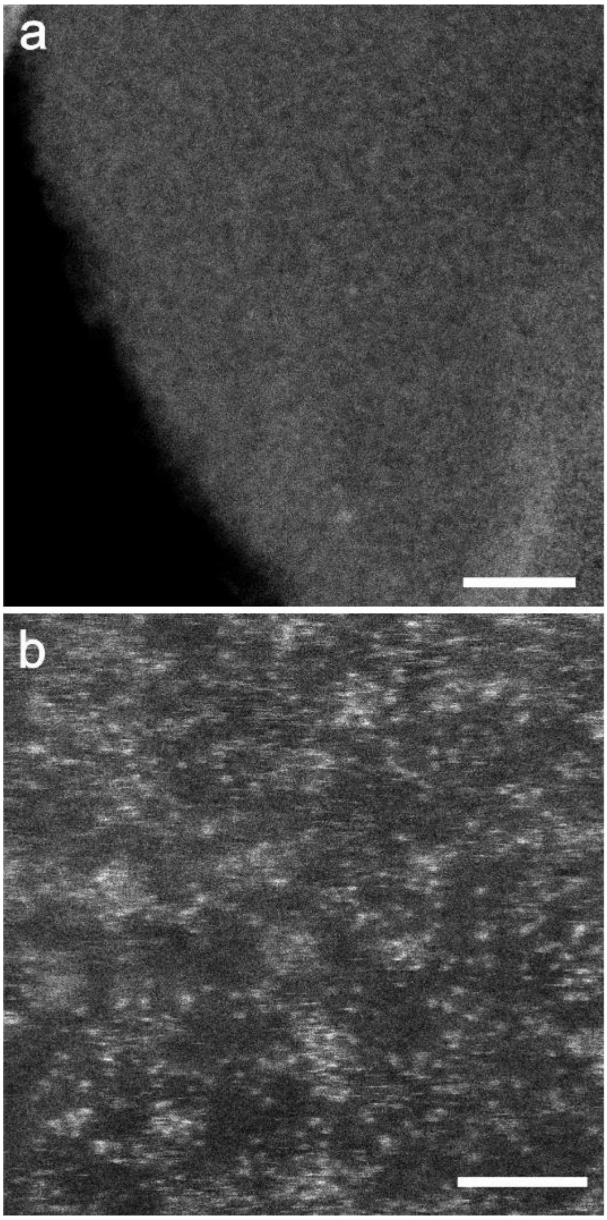

Supplementary Figure 9 | Representative aberration-corrected STEM-ADF images of Co_1/G-2.5 at different locations. Scale bars, 50 nm (a), 2nm (b).

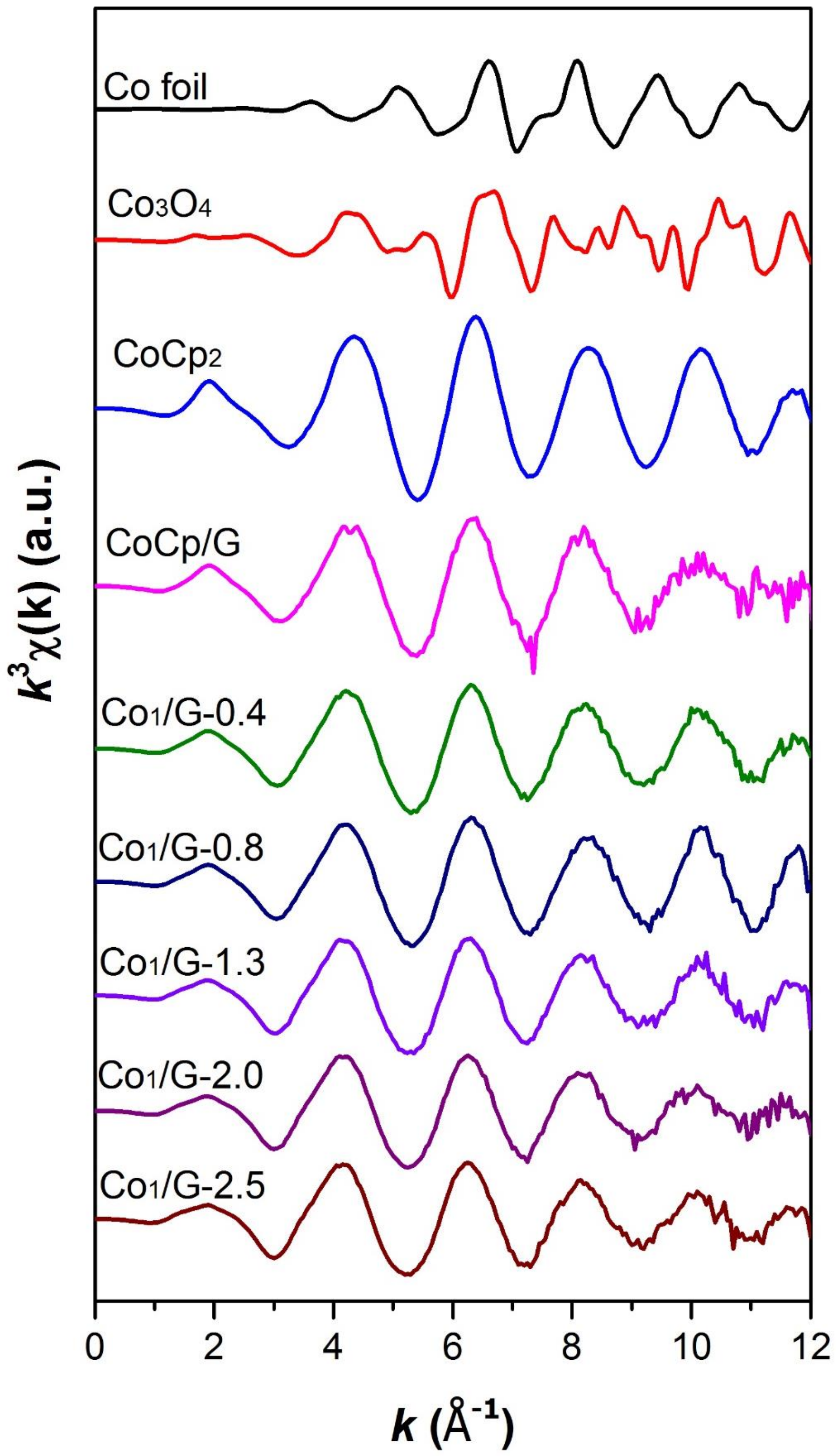

Supplementary Figure 10 | k^3-weighted EXAFS oscillations [$k^3\chi(k)$] of the CoCp/G and Co_1/G SACs at the Co *K*-edge. The spectra of Co foil, Co_3O_4 and $CoCp_2$ reference samples are also shown for comparison.

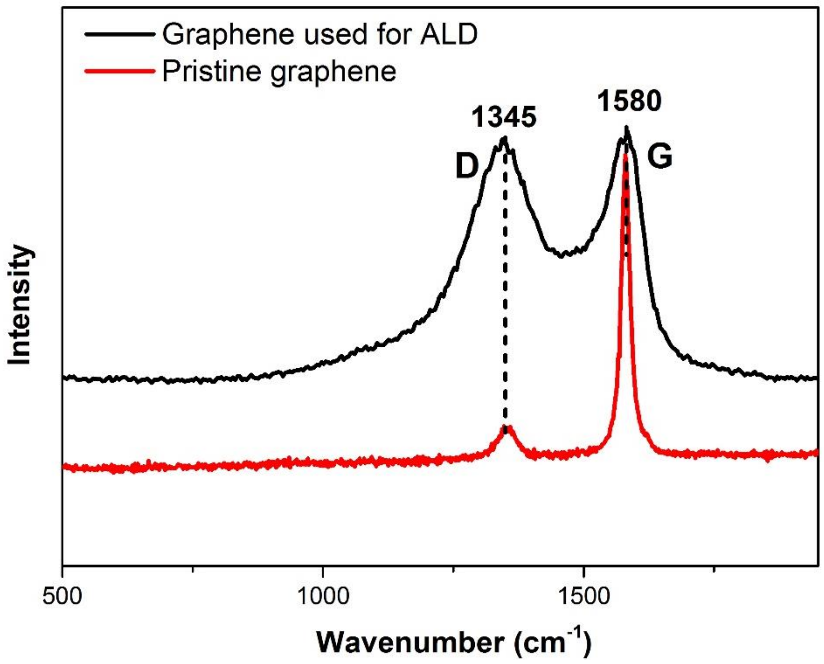

Supplementary Figure 11 | Raman spectra of the as-received graphene flakes and graphene oxide support. Note: The high ratio of D band to G band implies the coexistence of small domains of the sp^2 carbons along with a high density of vacancy- or oxygen-related defects in the graphene oxide.[4]

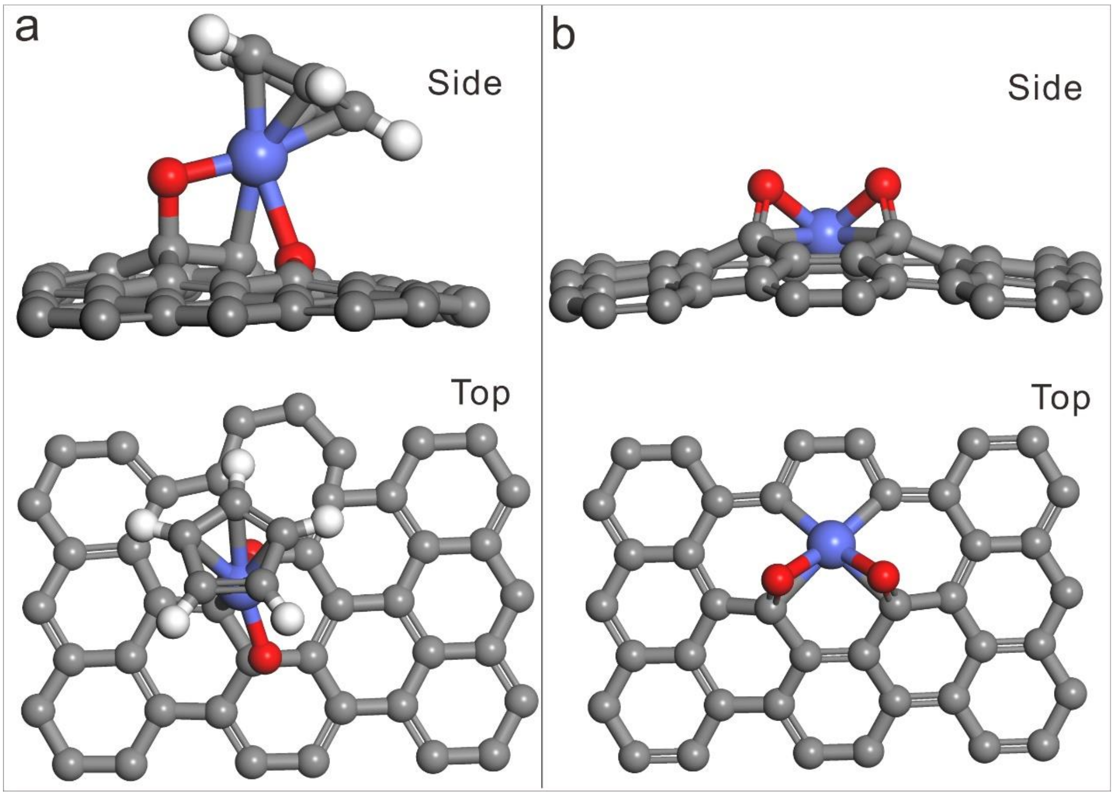

Supplementary Figure 12 │The optimized model for CoCp/G (a) and Co_1/G SACs (b) by DFT calculations. The balls in grey, white, red and blue represent carbon, hydrogen, oxygen and cobalt atoms.

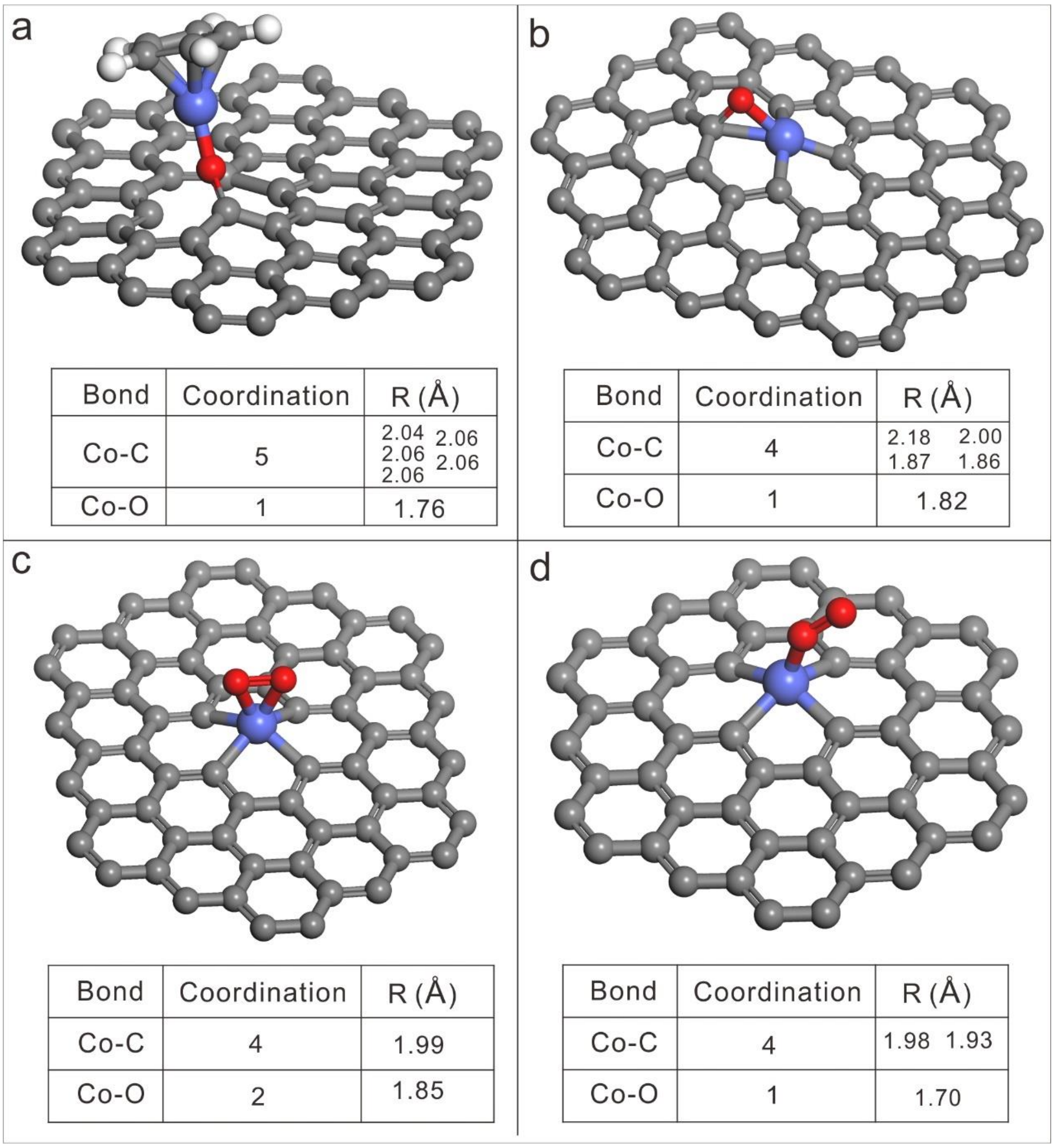

Supplementary Figure 13 | DFT calculations of the proposed atomic structures of CoCp/G (a) and Co_1/G SACs (b, c, d). The balls in grey, white, red and blue represent carbon, hydrogen, oxygen and cobalt.

Notes: The proposed Co_1 structure consisting of one interfacial oxygen (b) or adsorbed dioxygen (c,d) exhibits a much shorter Co-O bond compared with the bonding length (1.94 Å) extracted from EXAFS fitting. Hence, the structures proposed above for Co_1/G SACs can be safely excluded.

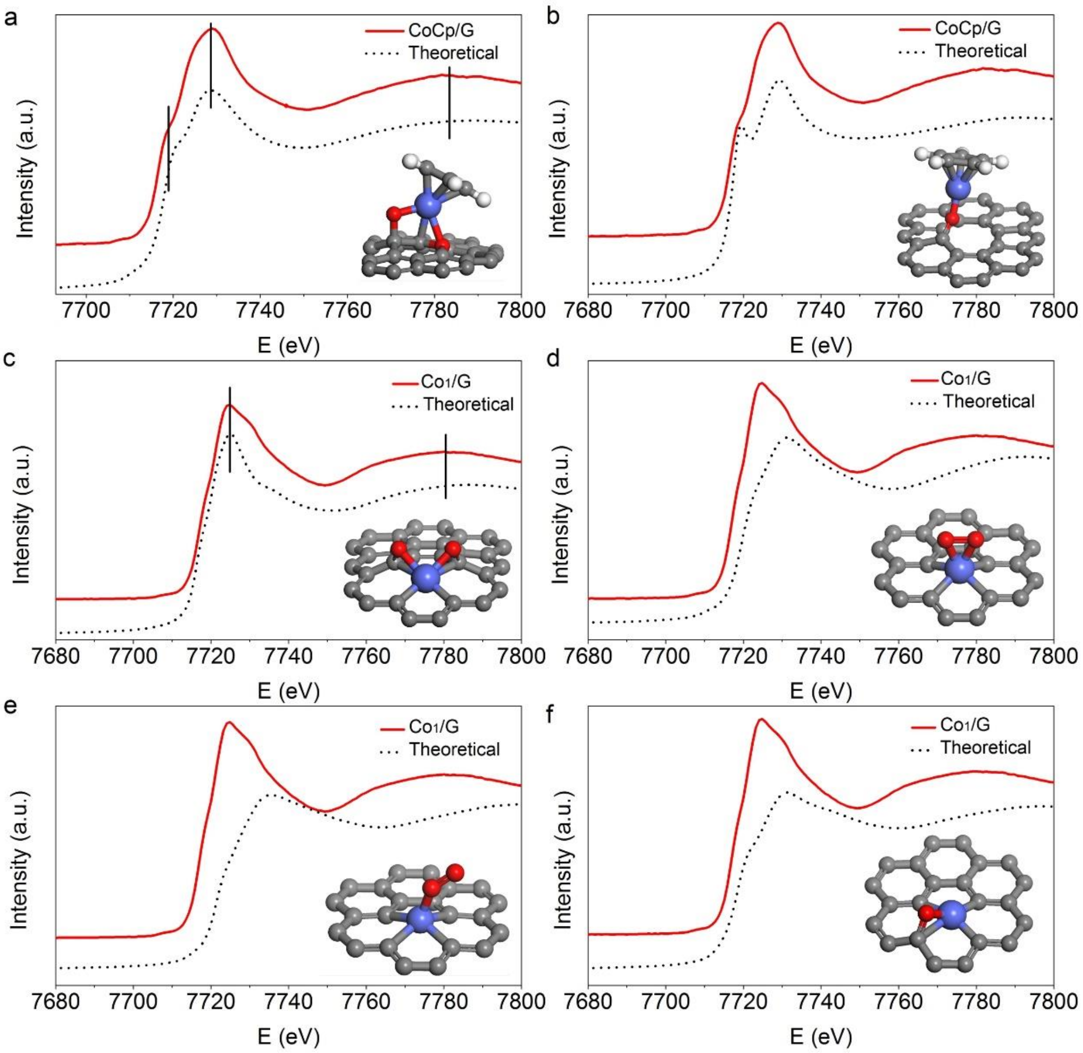

Supplementary Figure 14 │ The comparison of experimental and modelled XANES spectra of DFT-modelled structures (the corresponding atomic strucures are shown in the inset).

Supplementary Table 1 | The comparison of EXAFS Fitting results and the optimized model of CoCp/G and Co_1/G samples.

EXAFS fitting				
**Sample**	**Shell**	***R*/Å**	***N***	**σ^2 x 10^3($Å^2$)**
$CoCp_2$	Co-C	2.04	10.0	2.6
CoCp/G	Co-O	1.95	2.7	0.9
	Co-C	2.10	4.8	1.7
Co_1/G	Co-O	1.94	2.7	1.4
	Co-C	2.09	4.2	1.4

DFT			
**Sample**	**Shell**	***R*/Å**	***N***
CoCp/G	Co-O	2.00	2
	Co-C	2.05	6
Co_1/G	Co-O	1.90	2
	Co-C	2.03	4

N, coordination numbers; R, bonding distance; σ^2, Debey-Waller factor. Errors in the fitting parameters are N ±20%, R ± 0.02 Å, σ^2 ±20%.

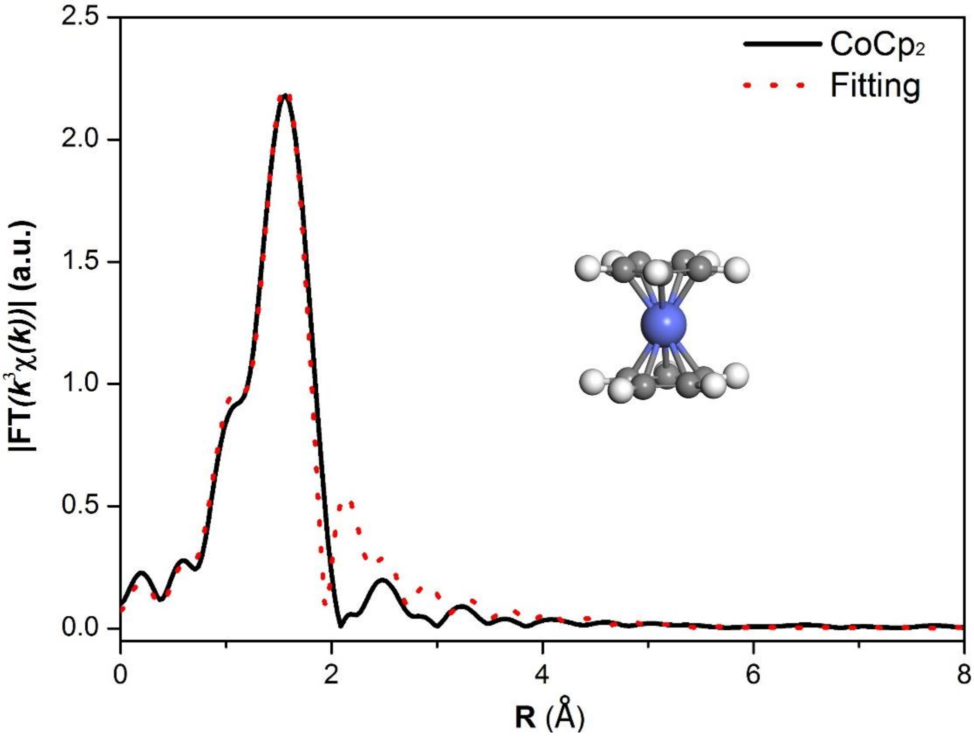

Supplementary Figure 15 | Fourier transform (FT) extended x-ray absorption fine structure (EXAFS) and fitting curve for $CoCp_2$ with the corresponding structures modelled by DFT calculations (insets).

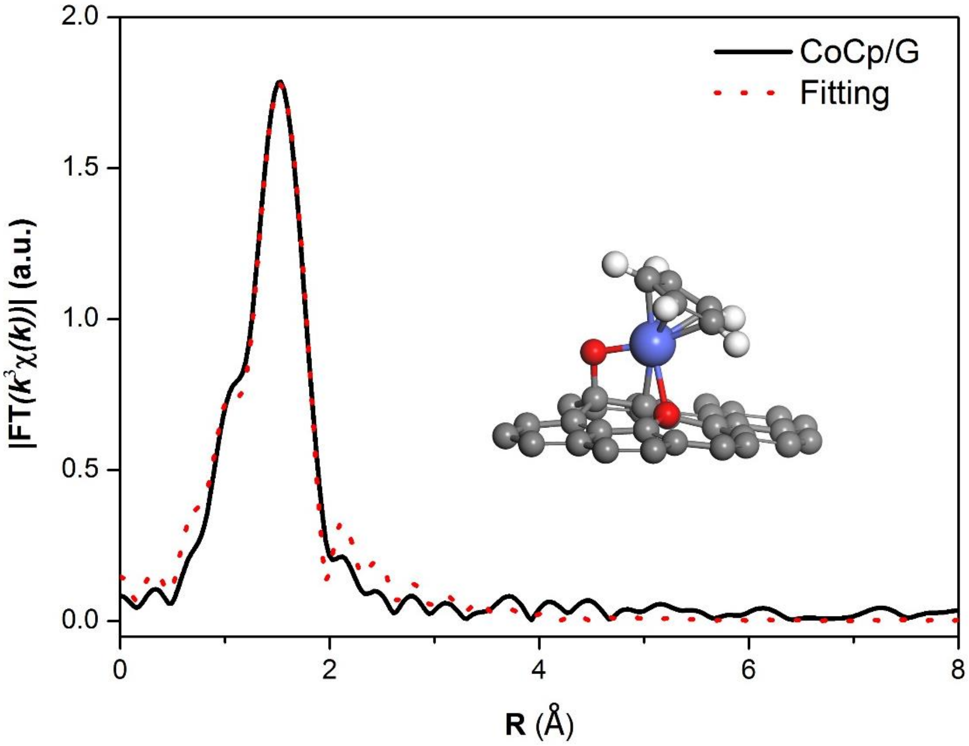

Supplementary Figure 16 | Fourier transform (FT) extended x-ray absorption fine structure (EXAFS) and fitting curve for CoCp/G with the corresponding structures modelled by DFT calculations (insets).

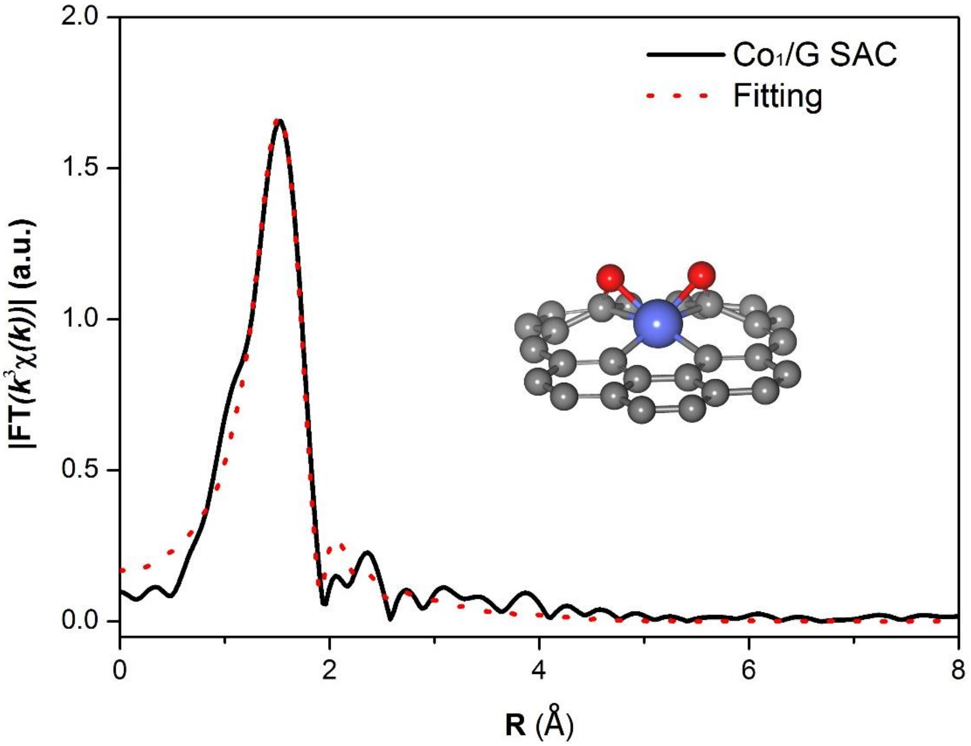

Supplementary Figure 17 | Fourier transform (FT) extended x-ray absorption fine structure (EXAFS) and fitting curve for Co_1/G SAC with the corresponding structures modelled by DFT calculations (insets).

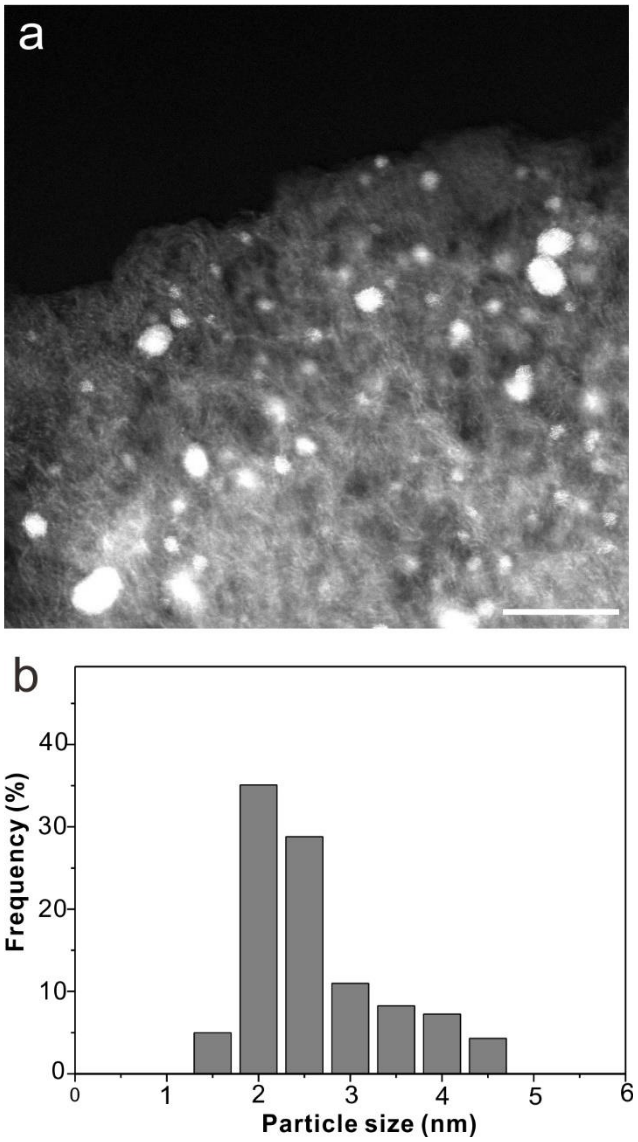

Supplementary Figure 18 | Morphology of the commercial Pt/carbon catalyst. (**a**) STEM-ADF image of the Pt/carbon sample. (**b**) The histogram of the Pt particle size distribution. The average size of Pt nanoparticles is determined to be about 2.6 nm. Scale bars, 10 nm (a).

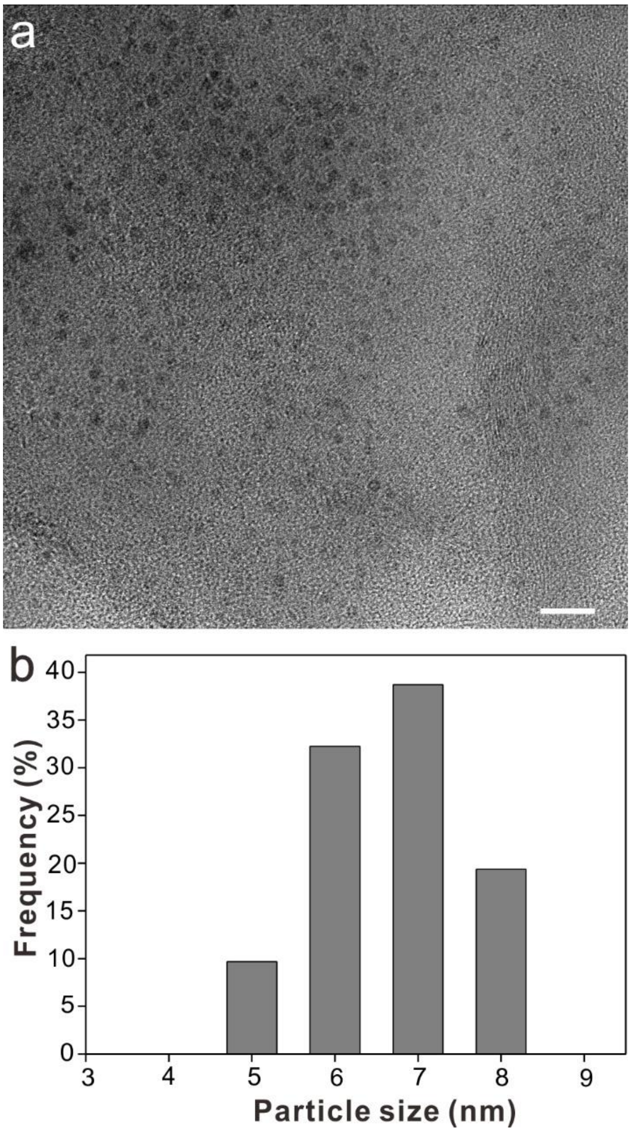

Supplementary Figure 19 | Morphology of the obtained Co-NPs/G catalyst. (**a**) TEM image of the Co-NPs/G sample. (**b**) The histogram of the size distribution of Co nanoparticles. The average size of Co particles is determined to be about 6.7 nm. Sacle bars, 20 nm (a).

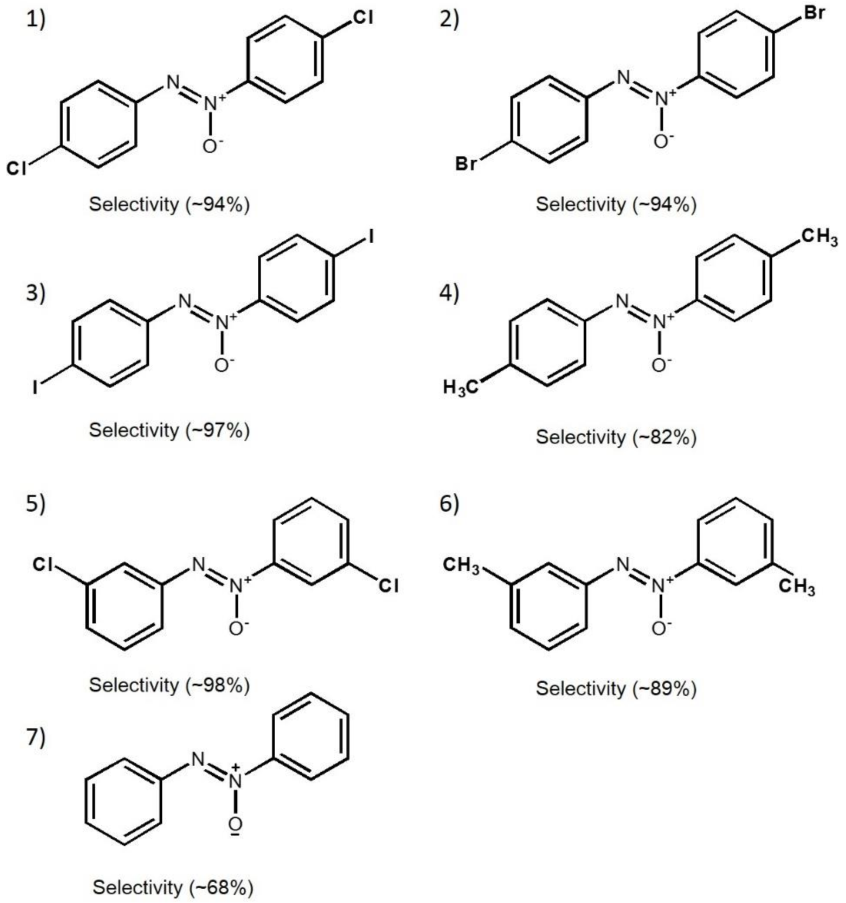

Supplementary Figure 20 | Selectivity to azoxy compounds for different substrates over all the Co_1/G SACs.

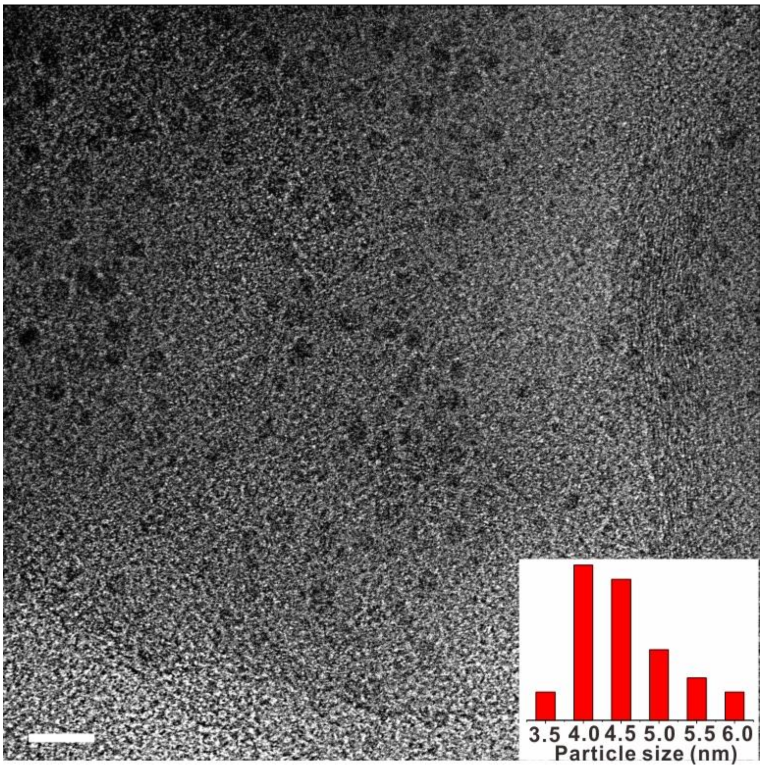

Supplementary Figure 21 | Morphology of the obtained Co-NPs/G-ALD catalyst. The TEM image of the Co-NPs/G-ALD sample and size distribution of Co nanoparticles (inset). The average size of Co particles is determined to be about 4.3 nm. Scale bars, 10 nm.

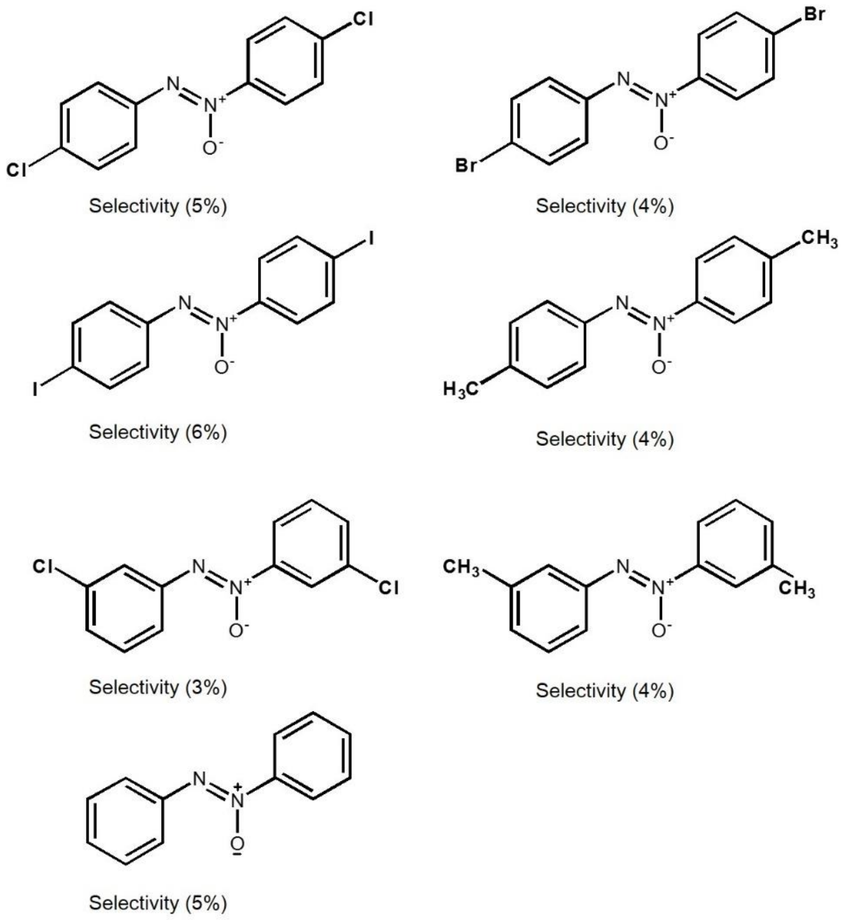

Supplementary Figure 22 | Selectivity to azoxy compounds for different substrates over Co-NPs/G-ALD.

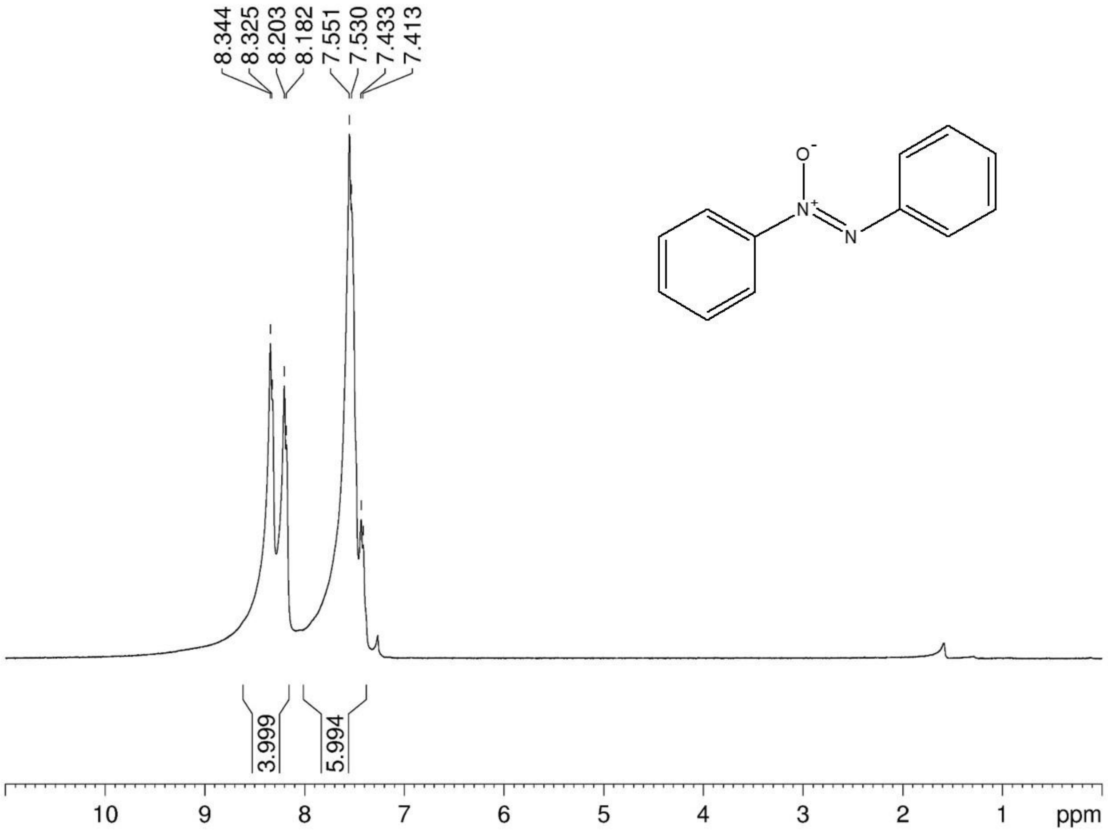

Supplementary Figure 23 | ^{1}H NMR spectrum of Azoxybenzene. The residual solvent peak was used as an internal reference: ^{1}H (chloroform, 300 MHz). δ 7.41–7.55 (m, 6 H, ArH), 8.18–8.20 (d, 2 H, ArH), 8.32–8.34 (d, 2 H, ArH)

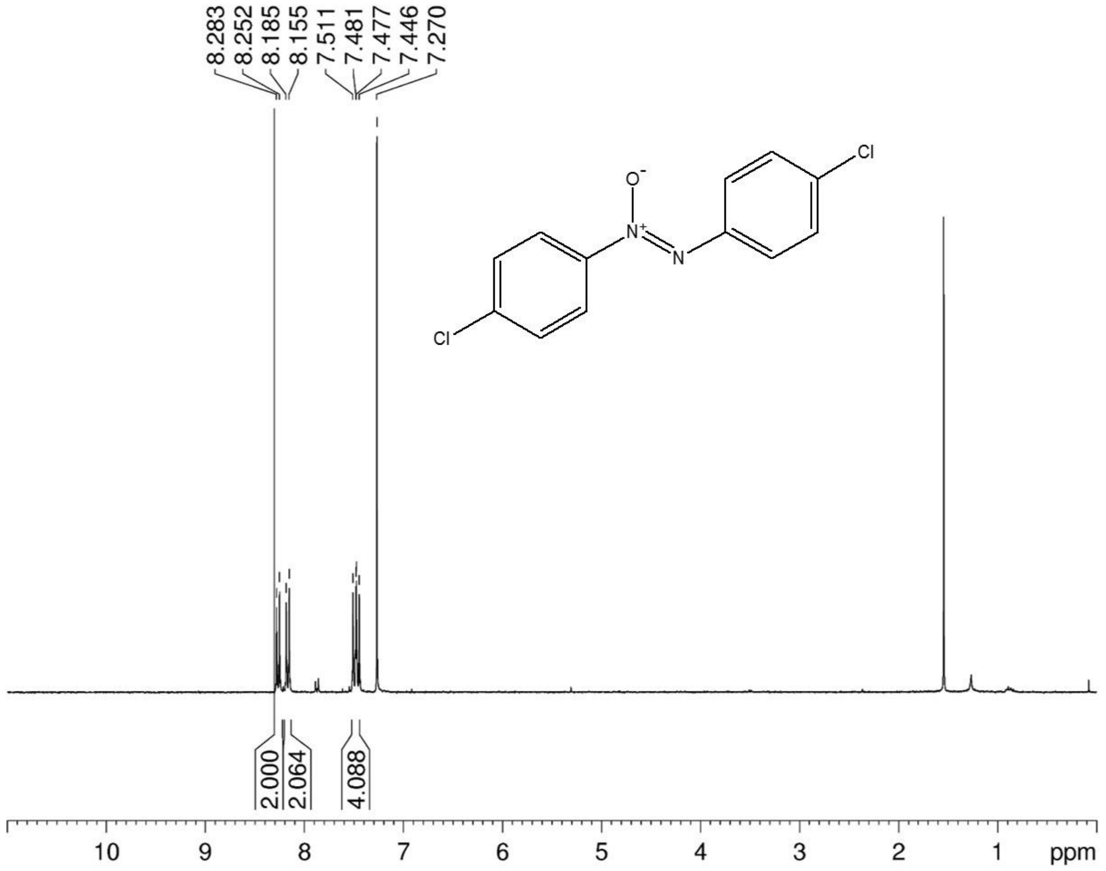

Supplementary Figure 24 | ^{1}H NMR spectrum of 4, 4'-dichloride azoxybenzene. The residual solvent peak was used as an internal reference: ^{1}H (chloroform, 300 MHz). δ 7.45–7.51 (m, 4 H, ArH), 8.15–8.19 (d, 2 H, ArH), 8.25–8.28 (d, 2 H, ArH)

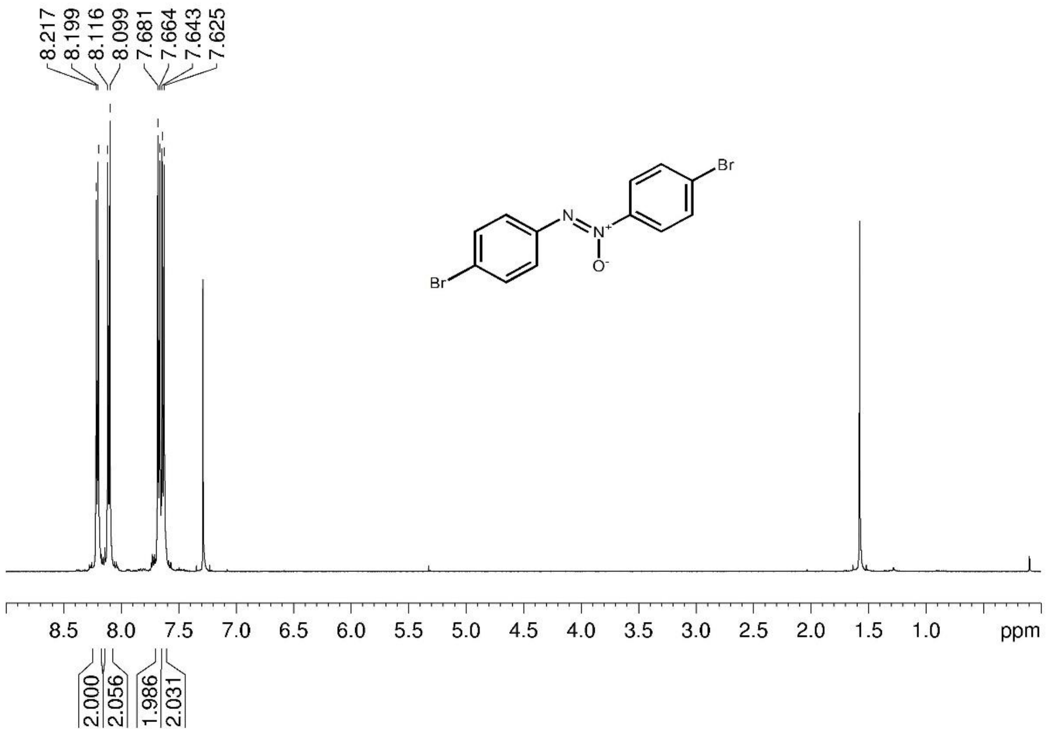

Supplementary Figure 25 | ^{1}H NMR spectrum of 4, 4'-dibromo azoxybenzene. The residual solvent peak was used as an internal reference: ^{1}H (chloroform, 500 MHz). δ 7.62–7.64 (d, 2H, ArH), 7.66–7.68 (d, 2 H, ArH), 8.10–8.12 (d, 2 H, ArH), 8.20–8.22 (d, 2 H, ArH).

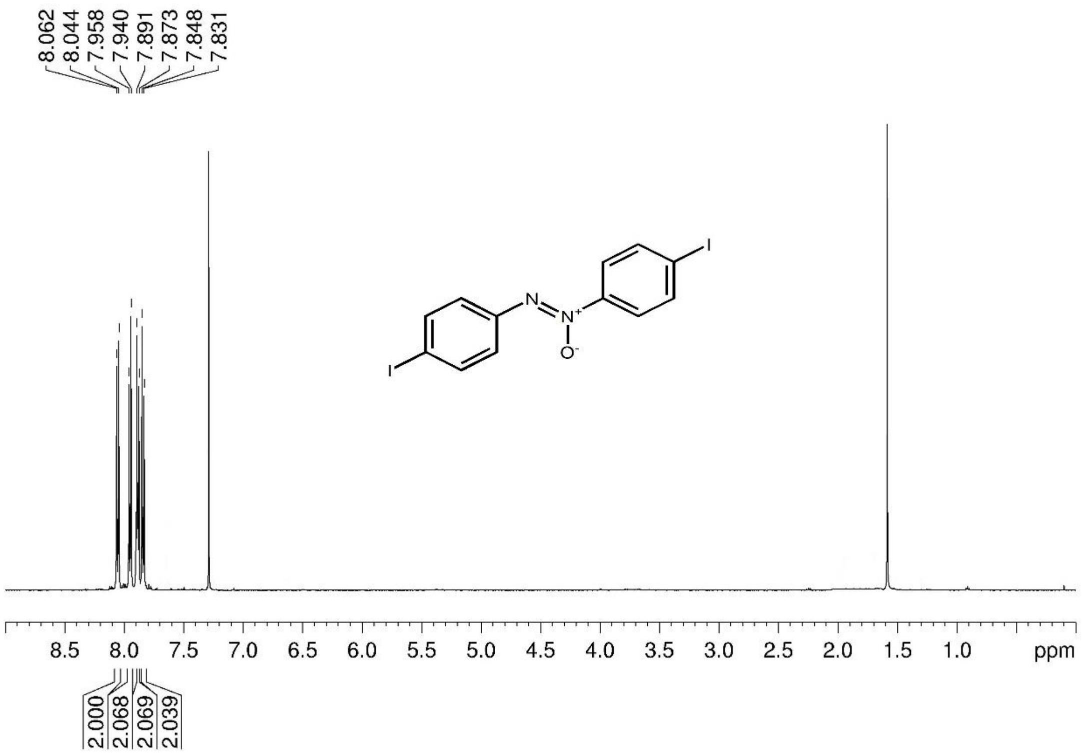

Supplementary Figure 26 | ^{1}H NMR spectrum of 4, 4'-diiodo azoxybenzene.

The residual solvent peak was used as an internal reference: ^{1}H (chloroform, 500 MHz). δ 7.83–7.85 (d, 2H, ArH), 7.87–7.89 (d, 2 H, ArH), 7.94–7.96 (d, 2 H, ArH), 8.04–8.06 (d, 2 H, ArH).

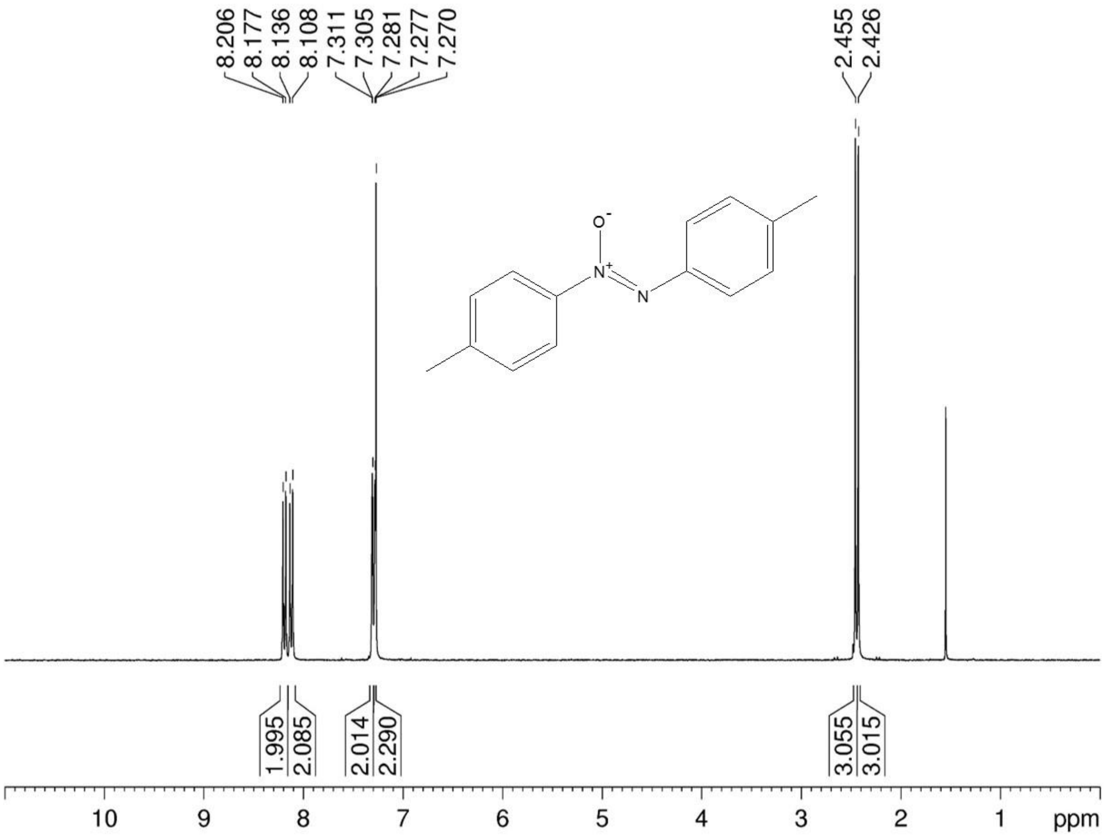

Supplementary Figure 27 | ^{1}H NMR spectrum of 4, 4'-dimethyl azoxybenzene. The residual solvent peak was used as an internal reference: ^{1}H (chloroform, 300 MHz). δ 2.43 (s, 3 H, CH_3), δ 2.46 (s, 3 H, CH_3), δ 7.28–7.31 (m, 4 H, ArH), 8.10–8.14 (d, 2 H, ArH), 8.17–8.21 (d, 2 H, ArH)

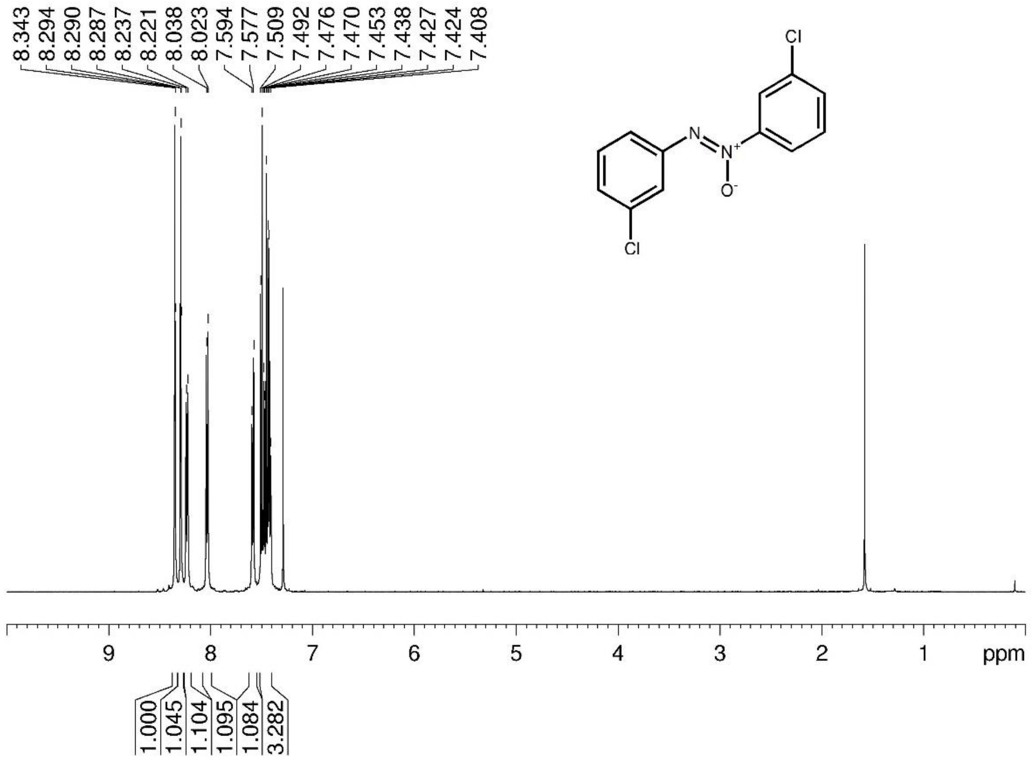

Supplementary Figure 28 | ^{1}H NMR spectrum of 3, 3'-dichloride azoxybenzene. The residual solvent peak was used as an internal reference: ^{1}H (chloroform, 500 MHz). δ 7.40–7.51 (m, 3H, ArH), 7.57–7.59 (d, 1H, ArH), 8.02–8.04 (d, 1 H, ArH), 8.22–8.34 (m, 3 H, ArH).

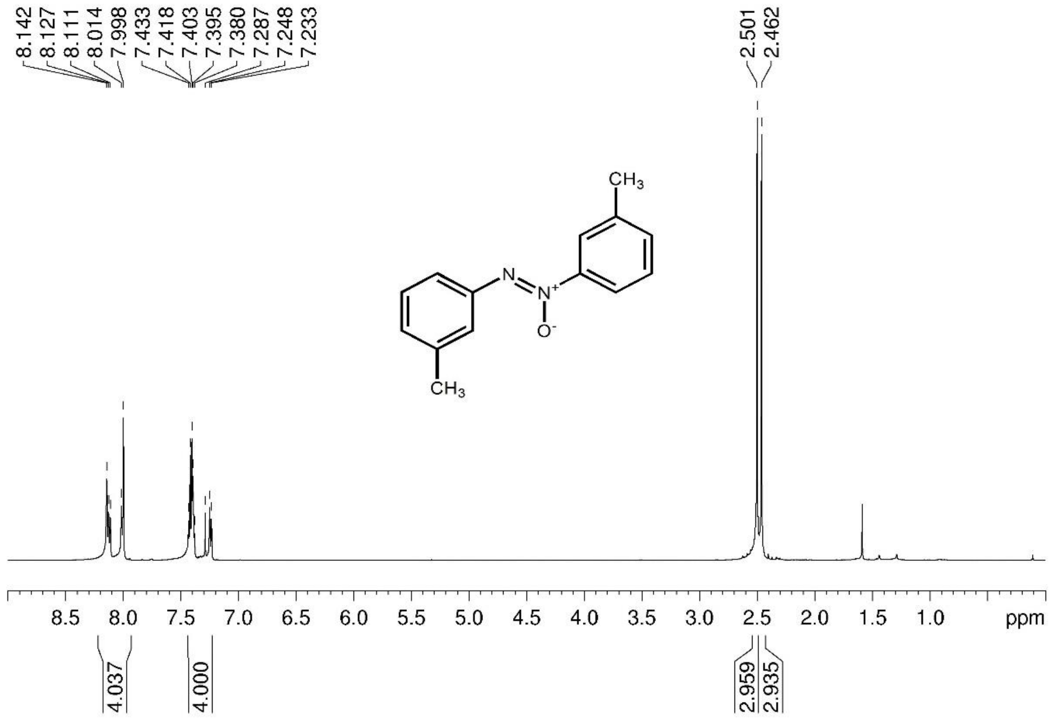

Supplementary Figure 29 | ^{1}H NMR spectrum of 3, 3'-dimethyl azoxybenzene. The residual solvent peak was used as an internal reference: ^{1}H (chloroform, 500 MHz). δ 2.46 (s, 3 H, CH_3), δ 2.50 (s, 3 H, CH_3), δ 7.23–7.43 (m, 4 H, ArH), 7.99–8.14 (m, 4H, ArH).

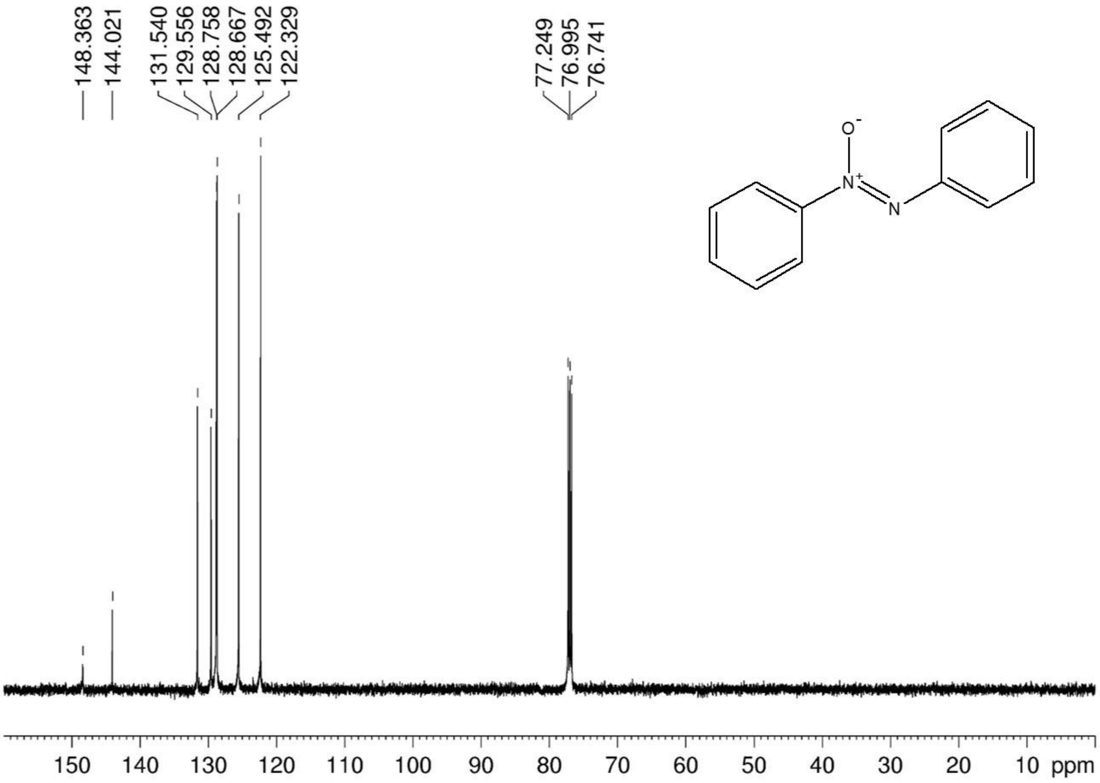

Supplementary Figure 30 | ^{13}C NMR spectrum of azoxybenzene. The residual solvent peak was used as an internal reference: ^{13}C (chloroform, 125 MHz). δ 122.33, 125.49, 128.67, 128.76, 129.56, 131.54, 144.02, 148.36.

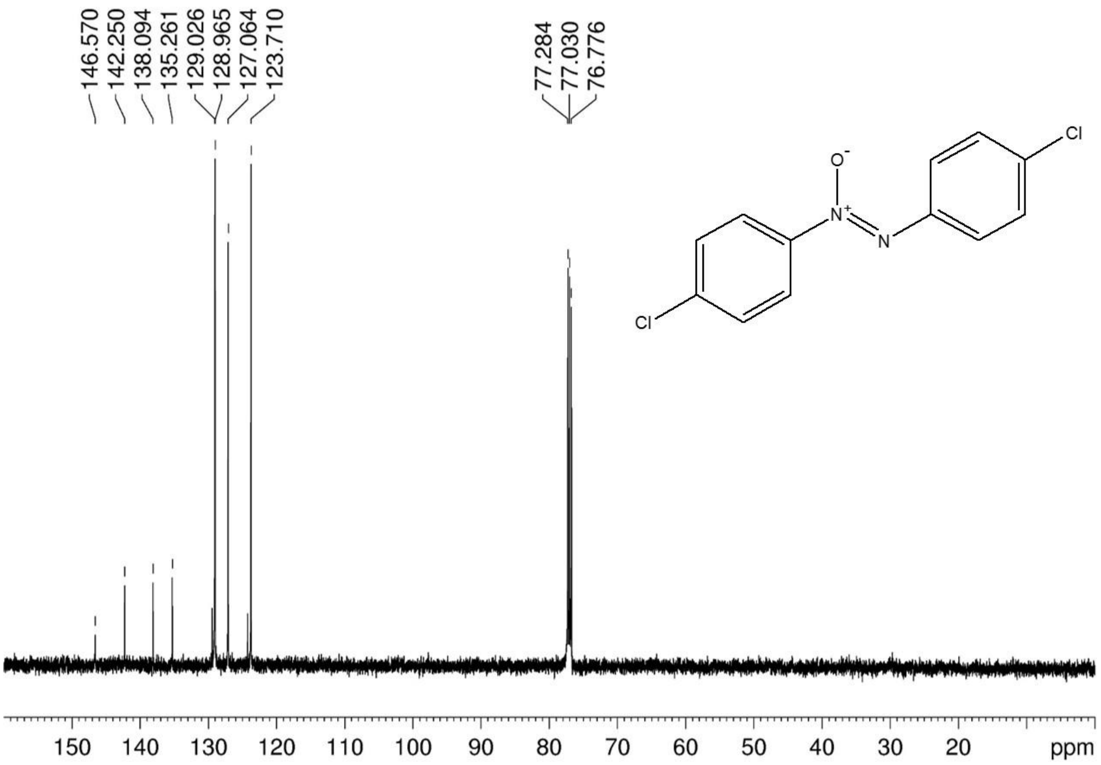

Supplementary Figure 31 | ^{13}C NMR spectrum of 4, 4'-dichloride azoxybenzene. The residual solvent peak was used as an internal reference: ^{13}C (chloroform, 125 MHz). δ 123.70, 127.06, 128.96, 129.03, 135.26, 138.09, 142.25, 146.57.

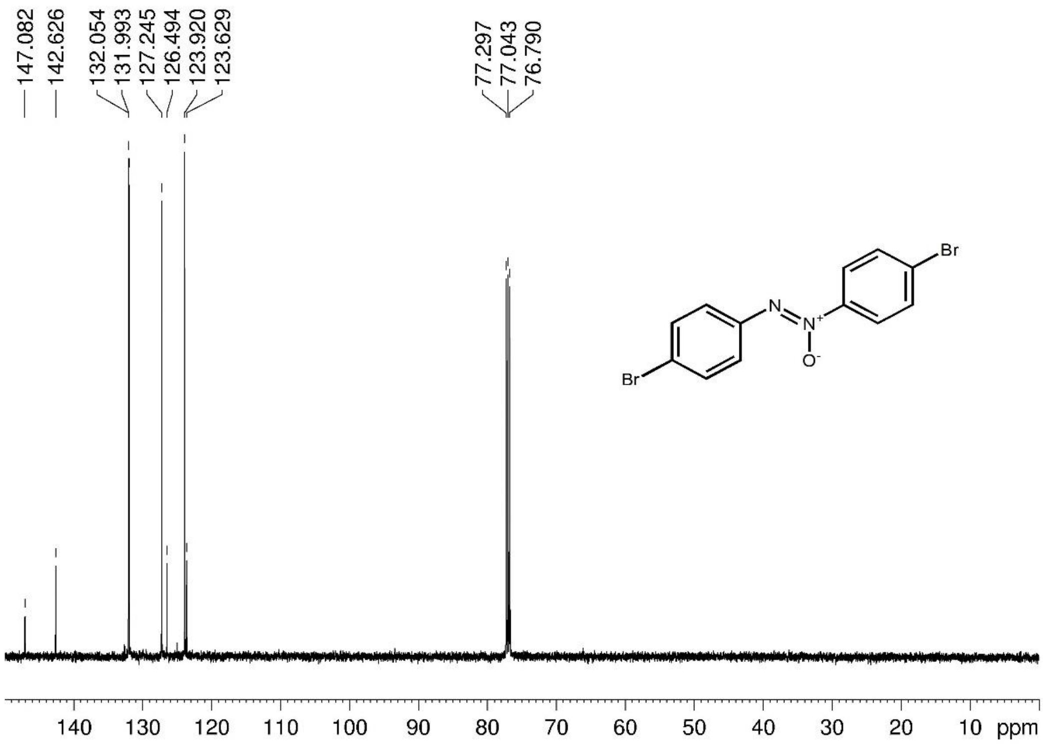

Supplementary Figure 32 | ^{13}C NMR spectrum of 4, 4'-dibromo azoxybenzene. The residual solvent peak was used as an internal reference: ^{13}C (chloroform, 125 MHz). δ 123.63, 123.92, 126.49, 127.25, 131.99, 132.05, 142.63, 147.08.

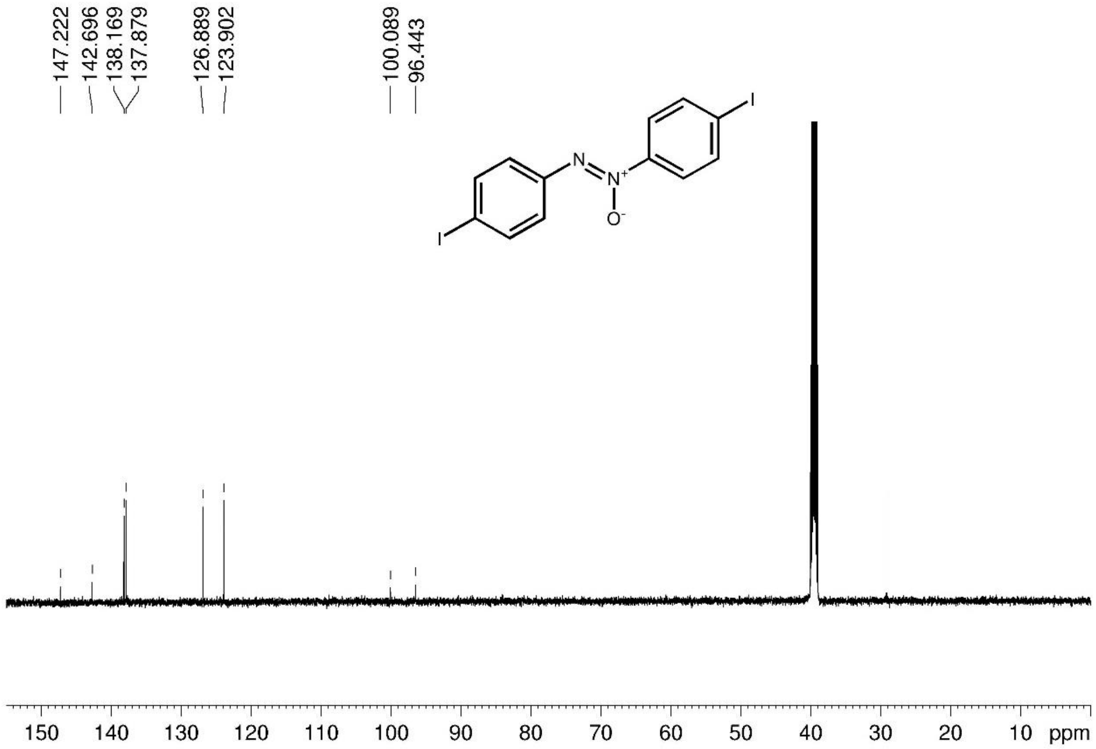

Supplementary Figure 33 | ^{13}C NMR spectrum of 4, 4'-diiodo azoxybenzene. The residual solvent peak was used as an internal reference: ^{13}C (DMSO, 125 MHz). δ 96.44, 100.09, 123.90, 126.89, 137.88, 138.17, 142.70, 147.22.

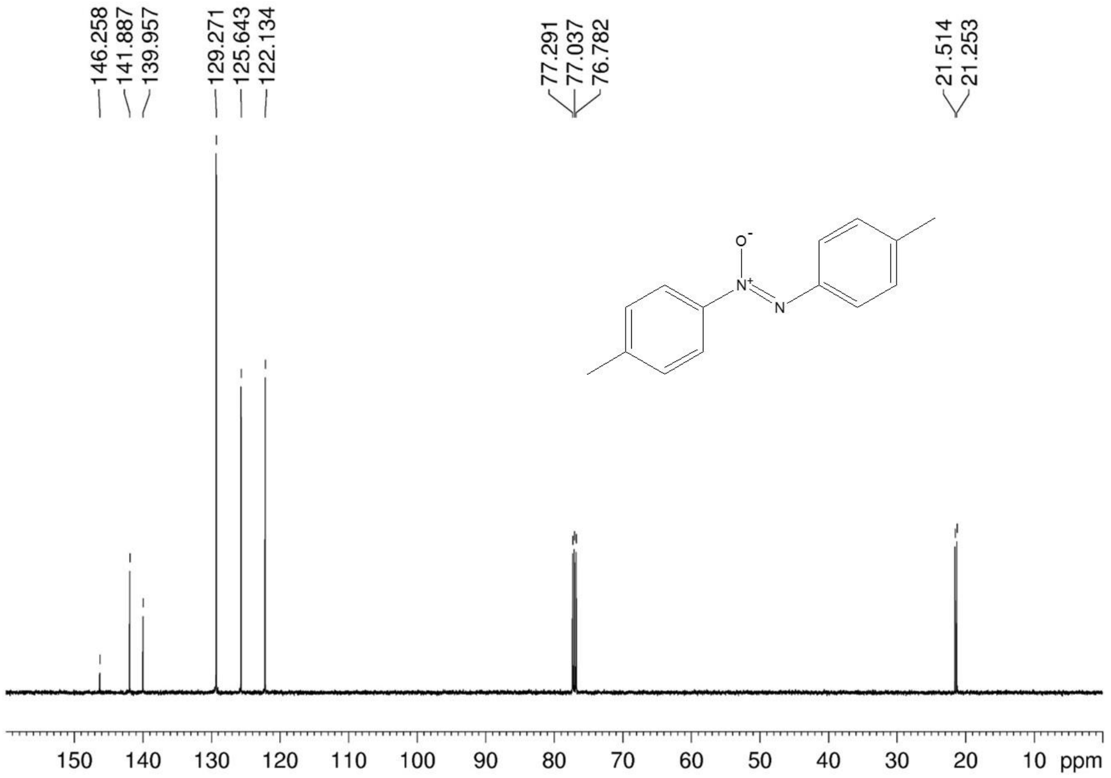

Supplementary Figure 34 | ^{13}C NMR spectrum of 4, 4'-dimethyl azoxybenzene. The residual solvent peak was used as an internal reference: ^{13}C (chloroform, 125 MHz). δ 21.25, 21.51, 122.13, 125.64, 129.27, 139.96, 141.89, 146.26.

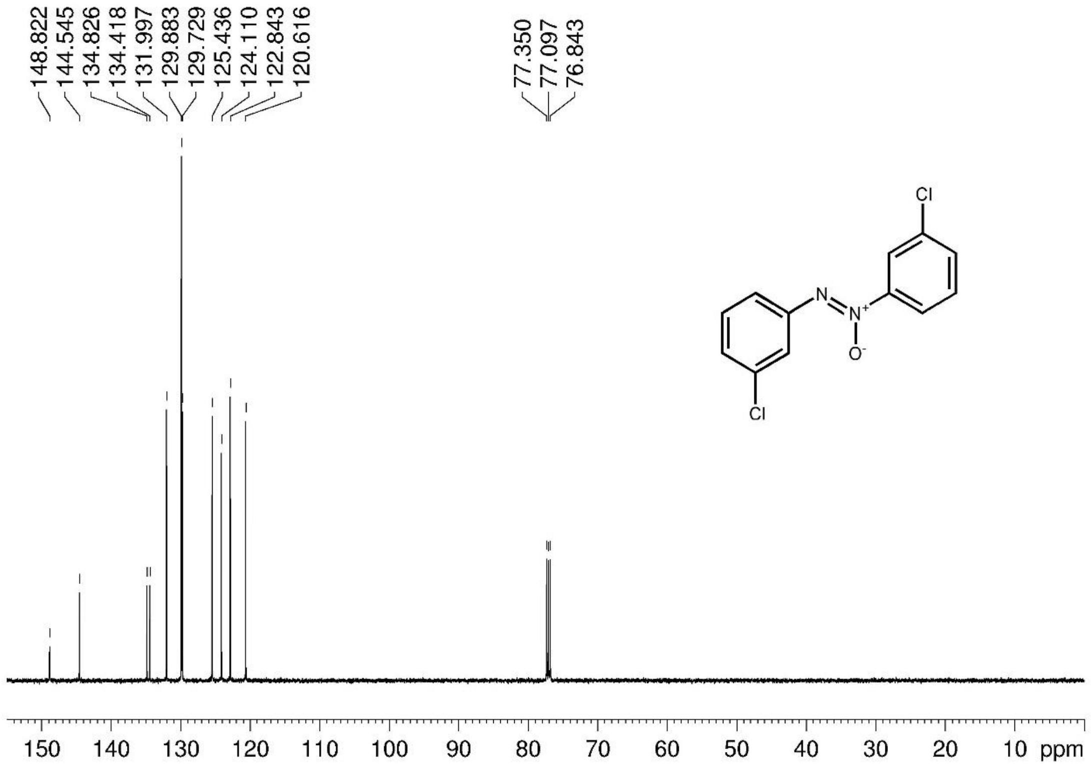

Supplementary Figure 35 | ^{13}C NMR spectrum of 3, 3'-dichloride azoxybenzene. The residual solvent peak was used as an internal reference: ^{13}C (chloroform, 125 MHz). δ 120.62, 122.84, 124.11, 125.44, 129.73, 129.88, 132.00, 134.42, 134.83, 144.55, 148.82.

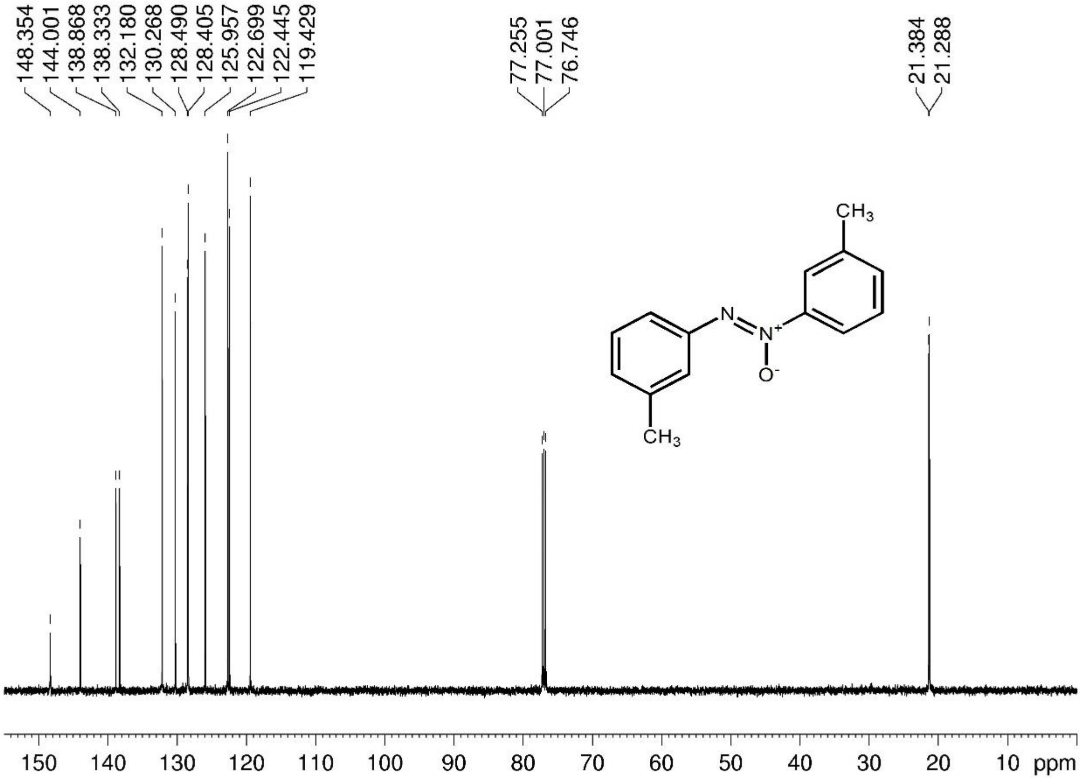

Supplementary Figure 36 | ^{13}C NMR spectrum of 3, 3'-dimethyl azoxybenzene. The residual solvent peak was used as an internal reference: ^{13}C (chloroform, 125 MHz). δ 21.29, 21.38, 119.43, 122.45, 122.70, 125.96, 128.41, 128.49, 130.27, 132.18, 138.33, 138.87, 144.00, 148.35.

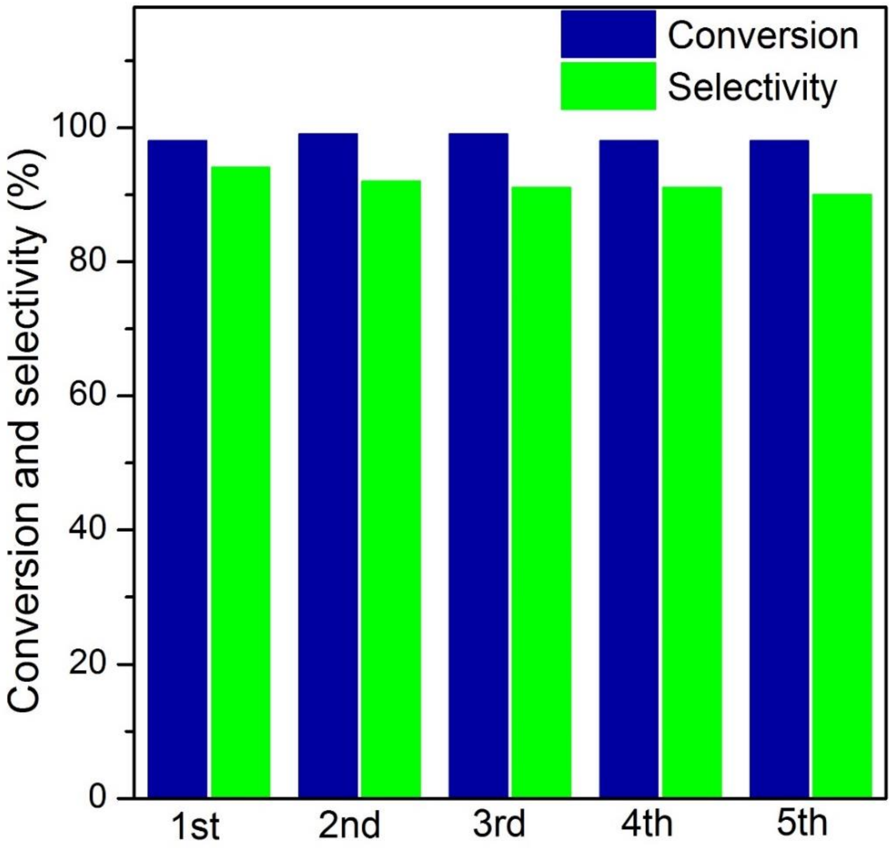

Supplementary Figure 37 | The nitrobenzene conversion and azoxybenzene selectivity over Co_1/G-2.5 SACs during five cycles in the hydrogenation of 1-chloride-4-nitrobenzene.

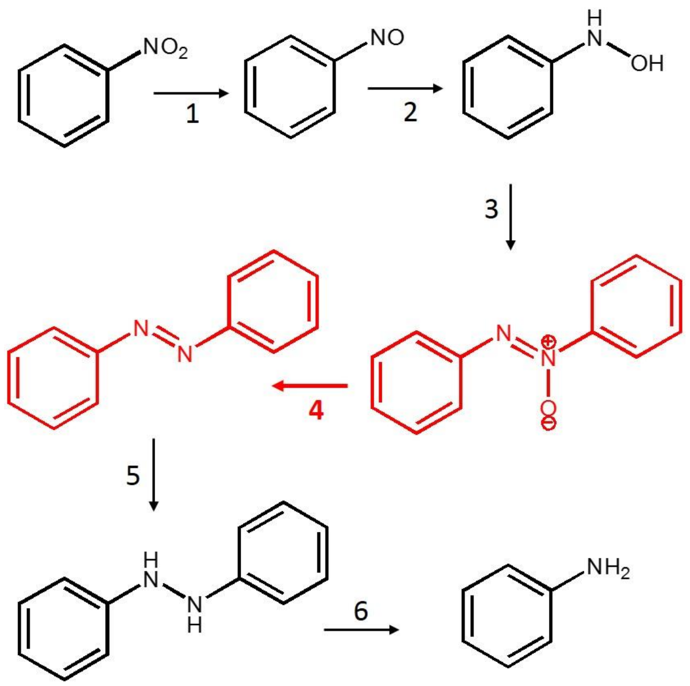

**Supplementary Figure 38 | ** The proposed reaction pathways of the hydrogenation of nitrobenzene. [5-7]

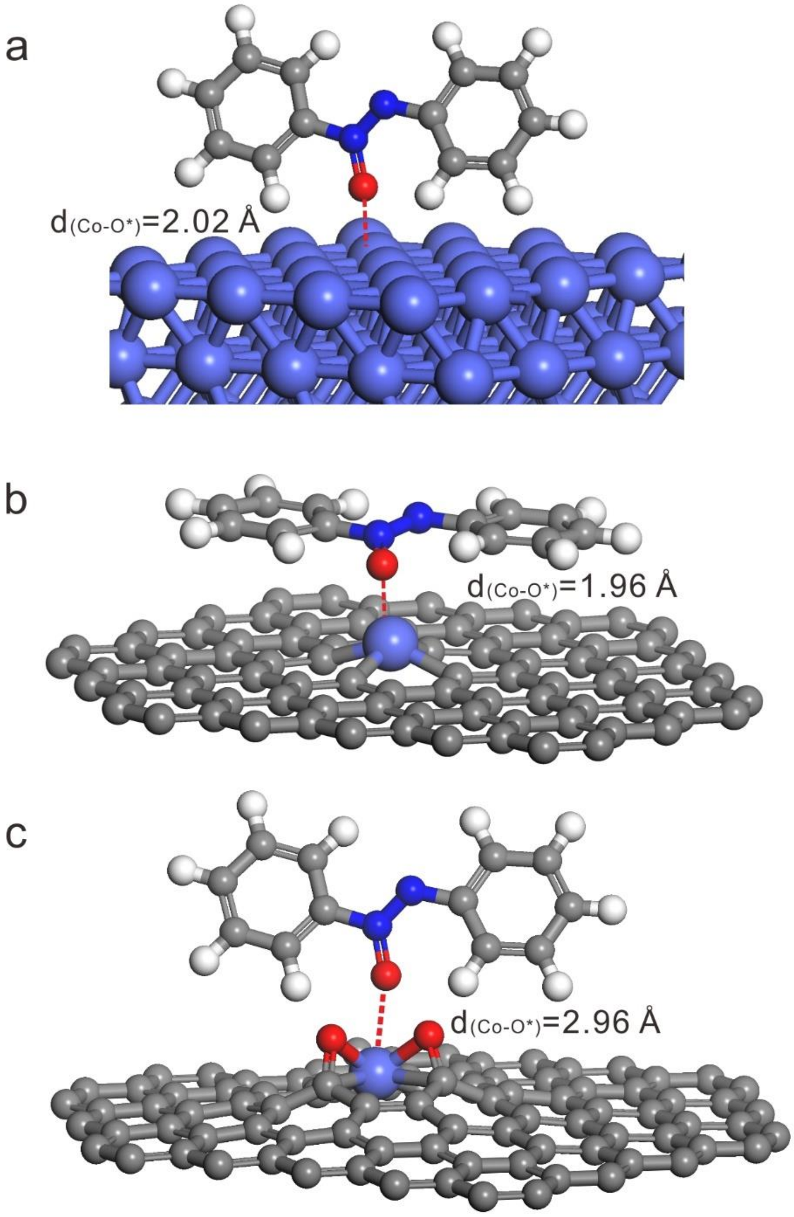

Supplementary Figure 39 | The calculated separation between the Co atom and the oxygen atom (labelled as $d_{(Co\text{-}O^*)}$) of azoxybenzene in the case of Co (111) (a), Co_1-C_4/G (b) and Co_1/G (c).

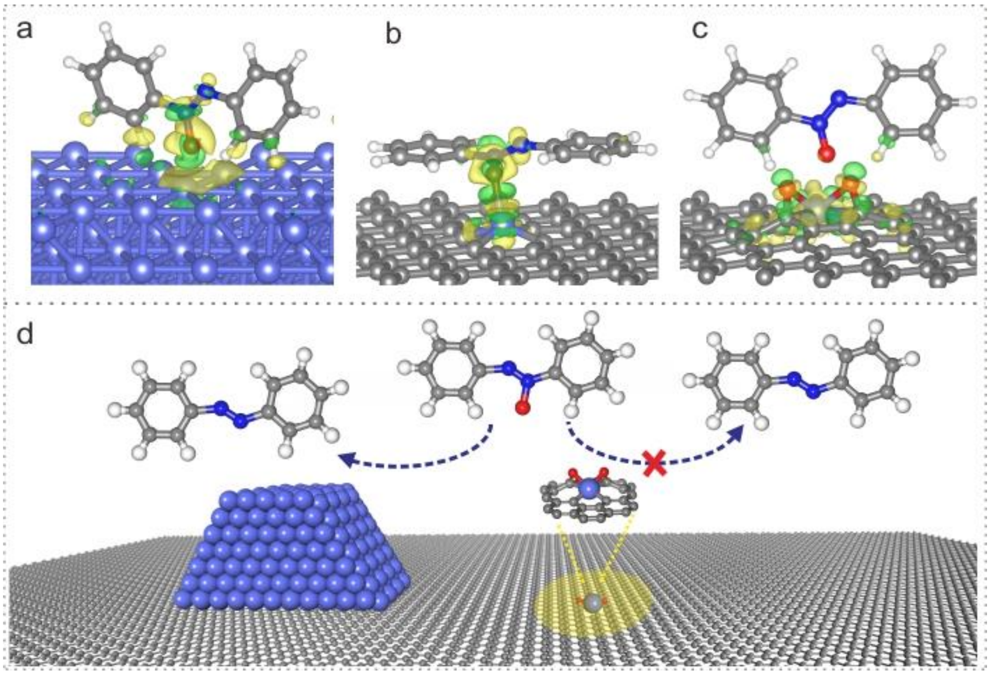

Supplementary Figure 40 | The charge transfer analyses for azoxybenzene molecule on Co (111) facet (a), Co_1-C_4/G (b) and Co_1/G (c). (d) Schematic illustration of the hydrogenation of azoxybenzene molecules on the Co nanoparticle and Co_1 single atom**.** Green color represents gaining electrons and yellow color represents losing electrons. The same isosurface values are chosen for both systems. The balls in grey, white, red and blue represent carbon, hydrogen, oxygen and cobalt, respectively.

Supplementary Table 2 | A comparison of catalytic performances of various catalysts for hydrogenation of nitroarenes.

Sample	Size (nm)	TOF (s^{-1})	Note
**Co_1/G-0.4**	-	**0.33**	**This work**
**Co_1/G-0.8**	-	**0.34**	**This work**
**Co_1/G-1.3**	-	**0.32**	**This work**
**Co_1/G-2.0**	-	**0.33**	**This work**
**Co_1/G-2.5**	-	**0.34**	**This work**
**Co-NPs/G**	**6.7**	**0.05**	**This work**
**Pt/C**	**2.6**	**0.25**	**This work**
Au/CeO_2	1.0	0.1	Ref [8]
Pd/graphene	8.0	0.3	Ref [9]
Pd/meso CdS	~5	0.3	Ref [10]
Pd/ND	5.0	1.4	Ref [11]
Pd NPs	3.0	0.53	Ref [12]
Pt/ND	5.0	0.48	Ref [11]
Ru/CMK-3	3.8	0.4	Ref [13]
Ru/C	3.8	0.15	Ref [13]
Rh/ Fe_3O_4@nSiO_2	3.0	0.9	Ref [14]
Rh@SiO_2/ Fe_3O_4@nSiO_2	3.0	1.2	Ref [14]
Fe-Ni NPs	-	0.05	Ref [15]
$Ni_{74}Sn_{26}$ alloy	4.5	0.01	Ref [16]
Ni/SiO_2	8.0	0.16	Ref [17]
Ni/G	-	0.01	Ref [18]
Cu NPs	50	0.09	Ref [19]

References:

1. Ganguly A, Sharma S, Papakonstantinou P, Hamilton J. Probing the thermal deoxygenation of graphene oxide using high-resolution in situ X-ray-based spectroscopies. *J. Phys. Chem. C* **115**, 17009-17019 (2011).
2. Yan H*, et al.* Single-atom Pd1/graphene catalyst achieved by atomic layer deposition: remarkable performance in selective hydrogenation of 1, 3-butadiene. *J. Am. Chem. Soc.* **137**, 10484-10487 (2015).
3. Krivanek OL*, et al.* Atom-by-atom structural and chemical analysis by annular dark-field electron microscopy. *Nature* **464**, 571-574 (2010).
4. Krishnamoorthy K, Veerapandian M, Yun K, Kim S-J. The chemical and structural analysis of graphene oxide with different degrees of oxidation. *Carbon* **53**, 38-49 (2013).
5. Zhu H, Ke X, Yang X, Sarina S, Liu H. Reduction of nitroaromatic compounds on supported gold nanoparticles by visible and ultraviolet light. *Angewandte Chemie* **122**, 9851-9855 (2010).
6. Hu L*, et al.* Highly efficient synthesis of aromatic azos catalyzed by unsupported ultra-thin Pt nanowires. *Chem. Commun.* **48**, 3445-3447 (2012).
7. Chen G*, et al.* Interfacial electronic effects control the reaction selectivity of platinum catalysts. *Nat Mater* **15**, 564 (2016).
8. Chong H*, et al.* Design of an ultrasmall Au nanocluster–CeO 2 mesoporous nanocomposite catalyst for nitrobenzene reduction. *Nanoscale* **5**, 7622-7628 (2013).
9. Tokai A*, et al.* One-pot preparation of Pd nanoparticles supported on graphene from Pd electrodes by discharge plasma in graphene suspension and its catalytic activity for hydrogenation of nitrobenzene. *Mater. Lett.* **199**, 24-27 (2017).
10. Zhou B*, et al.* Simultaneous and selective transformation of glucose to arabinose and nitrosobenzene to azoxybenzene driven by visible-light. *Green Chem.* **18**, 3852-3857 (2016).
11. Magdalinova N, Kalmykov P, Klyuev M. Hydrogenation catalysts based on platinum-and palladium-containing nanodiamonds. *Russian Journal of General Chemistry* **84**, 33-39 (2014).
12. Imura Y, Tsujimoto K, Morita C, Kawai T. Preparation and catalytic activity of Pd and bimetallic Pd–Ni nanowires. *Langmuir* **30**, 5026-5030 (2014).
13. Hu J, Ding Y, Zhang H, Wu P, Li X. Highly effective Ru/CMK-3 catalyst for selective reduction of nitrobenzene derivatives with H 2 O as solvent at near ambient temperature. *Rsc Adv.* **6**, 3235-3242 (2016).
14. Zhou J*, et al.* Porous Silica-Encapsulated and Magnetically Recoverable Rh NPs: a Highly Efficient, Stable and Green Catalyst for the Catalytic Transfer Hydrogenation with “Slow-Releasing” of Stoichiometric Hydrazine in Water. *Green Chem.*, (2017).
15. Petkar DR, Kadu BS, Chikate RC. Highly efficient and chemoselective transfer hydrogenation of nitroarenes at room temperature over magnetically separable Fe–Ni bimetallic nanoparticles. *Rsc Adv.* **4**, 8004-8010 (2014).
16. Shah M, Guo Q-X, Fu Y. The colloidal synthesis of unsupported nickel‑tin bimetallic nanoparticles with tunable composition that have high activity for the reduction of nitroarenes. *Catal. Commun.* **65**, 85-90 (2015).
17. Huang H*, et al.* Highly chemoselective reduction of nitroarenes over non-noble metal nickel-

molybdenum oxide catalysts. *Green Chem.* **19**, 809-815 (2017).

18. Pahalagedara MN*, et al.* Room temperature selective reduction of nitrobenzene to azoxybenzene over magnetically separable urchin-like Ni/Graphene nanocomposites. *J. Catal.* **336**, 41-48 (2016).

19. Kaur R, Giordano C, Gradzielski M, Mehta SK. Synthesis of Highly Stable, Water - Dispersible Copper Nanoparticles as Catalysts for Nitrobenzene Reduction. *Chem-Aisan J.* **9**, 189-198 (2014).
